# Developmental changes in the accessible chromatin, transcriptome and Ascl1-binding correlate with the loss in Müller Glial regenerative potential

**DOI:** 10.1038/s41598-020-70334-1

**Published:** 2020-08-12

**Authors:** Leah S. VandenBosch, Stefanie G. Wohl, Matthew S. Wilken, Marcus Hooper, Connor Finkbeiner, Kristen Cox, Laura Chipman, Thomas A. Reh

**Affiliations:** 1 Department of Biological Structure, University of Washington, Box 357420, Seattle, WA 98195 USA; 2 Molecular and Cellular Biology Program, University of Washington, Seattle, WA USA; 3 Institute for Stem Cells and Regenerative Medicine, University of Washington, Box 358056, Seattle, WA 98109 USA; 4 Department of Biological and Vision Sciences, College of Optometry, The State University of New York, New York, NY USA

**Keywords:** Epigenetic memory, Neurogenesis, Reprogramming, Transdifferentiation

## Abstract

Diseases and damage to the retina lead to losses in retinal neurons and eventual visual impairment. Although the mammalian retina has no inherent regenerative capabilities, fish have robust regeneration from Müller glia (MG). Recently, we have shown that driving expression of *Ascl1* in adult mouse MG stimulates neural regeneration. The regeneration observed in the mouse is limited in the variety of neurons that can be derived from MG; *Ascl1*-expressing MG primarily generate bipolar cells. To better understand the limits of MG-based regeneration in mouse retinas, we used ATAC- and RNA-seq to compare newborn progenitors, immature MG (P8-P12), and mature MG. Our analysis demonstrated developmental differences in gene expression and accessible chromatin between progenitors and MG, primarily in neurogenic genes. Overexpression of *Ascl1* is more effective in reprogramming immature MG, than mature MG, consistent with a more progenitor-like epigenetic landscape in the former. We also used ASCL1 ChIPseq to compare the differences in ASCL1 binding in progenitors and reprogrammed MG. We find that bipolar-specific accessible regions are more frequently linked to bHLH motifs and ASCL1 binding. Overall, our analysis indicates a loss of neurogenic gene expression and motif accessibility during glial maturation that may prevent efficient reprogramming.

## Introduction

The death of retinal neurons leads to permanent vision loss. While some species are readily capable of regenerating lost neurons, mammalian retinas are not. In mammals, neuron loss leads to reactive gliosis of the Müller glia (MG), similar to that of astrocytes in the brain^1^. Teleost fish, by contrast, are capable of regenerating retinal neurons, including photoreceptors and ganglion cells, after damage. This regeneration is carried out by the MG, which respond to damage by generating progenitor-like cells, similar to those in the developing retina^2,3^. Regeneration is accompanied by changes in gene expression and morphological changes to the MG, potentially regulated by epigenomic changes. The murine retina also undergoes epigenomic changes after damage, but neurogenic programs are not re-expressed, and neuronal regeneration does not occur^4^.

A critical difference between fish and mammalian MG in their response to damage is in their expression of the proneural transcription factor *Ascl1*. In fish, *Ascl1* is quickly upregulated after damage, and is necessary for regeneration of new neurons^5,6^. In the murine retina, *Ascl1* is expressed in retinal progenitors and necessary for development of rods and bipolar cells^7^; however it is not expressed in mature MG; moreover, after damage or in disease models, mouse MG do not spontaneously upregulate *Ascl1*^1,8^. We recently directed *Ascl1* expression to mouse MG with a inducible transgenic approach to test whether *Ascl1* expression is sufficient to induce regeneration. Expression of *Ascl1* in the MG of young mice (12 days post-natal (P12)) stimulated MG to generate new bipolar neurons after NMDA damage^8^. In adult mice, however, *Ascl1* over-expression in the MG is no longer sufficient to induce neurogenic potential, even in the presence of damage^9^. In mature mice, the addition of the histone deacetylase trichostatin-A (TSA), in combination with *Ascl1* overexpression and NMDA damage is required for neurogenesis; up to 30% of the *Ascl1*-expressing MG produce functional bipolar- and amacrine-like interneurons, confirmed by single cell transcriptomics, electrophysiology, and electron microscopy^9^.

The fact that the neurogenic program can be activated in mature MG by *Ascl1* only in combination with HDAC inhibition suggests that epigenetic mechanisms may limit regeneration from the MG. In addition, even with the addition of HDAC inhibitors, the *Ascl1*-expressing MG generate a subset of the neurons in the retina, suggesting that epigenetic factors may also limit the types of neurons that can be regenerated from mammalian MG^9^. Therefore, we asked whether changes in the transcriptome or epigenetic landscape might account for the difference in neurogenic potential between MG and retinal progenitors.

To address the question of what distinguishes mature MG from late progenitors, we performed a transcriptomic and epigenomic comparison of FACS-isolated postnatal progenitors, young MG (before eye-opening at P12), and adult MG. Analysis by ATAC and RNA sequencing demonstrates a clear trend in the loss of neurogenesis-related motif accessibility and expression. Immature MG are found to have an epigenomic and transcriptomic profile that is intermediate between mature MG and progenitors. To test whether the intermediate profile of young MG correlates with their neurogenic potential, we over-expressed *Ascl1* in developing MG. We identified key restriction points in the neurogenic potential of MG that correlate with changes in the accessible chromatin landscape. To better understand the role of the bHLH factor *Ascl1* in driving retinal regeneration from MG, we performed ASCL1 ChIP-seq on P0 retinas, and on MG following *Ascl1* overexpression. Interestingly, bipolar-specific accessible regions are enriched in bHLH motifs and ASCL1 binding in reprogrammed MG when compared with P2 progenitors. Our results thus indicate a loss of neurogenic genes and their accessible motifs during MG maturation that may have implications for regeneration.

## Results

### Chromatin accessibility in retinal progenitors

**Figure 1:**
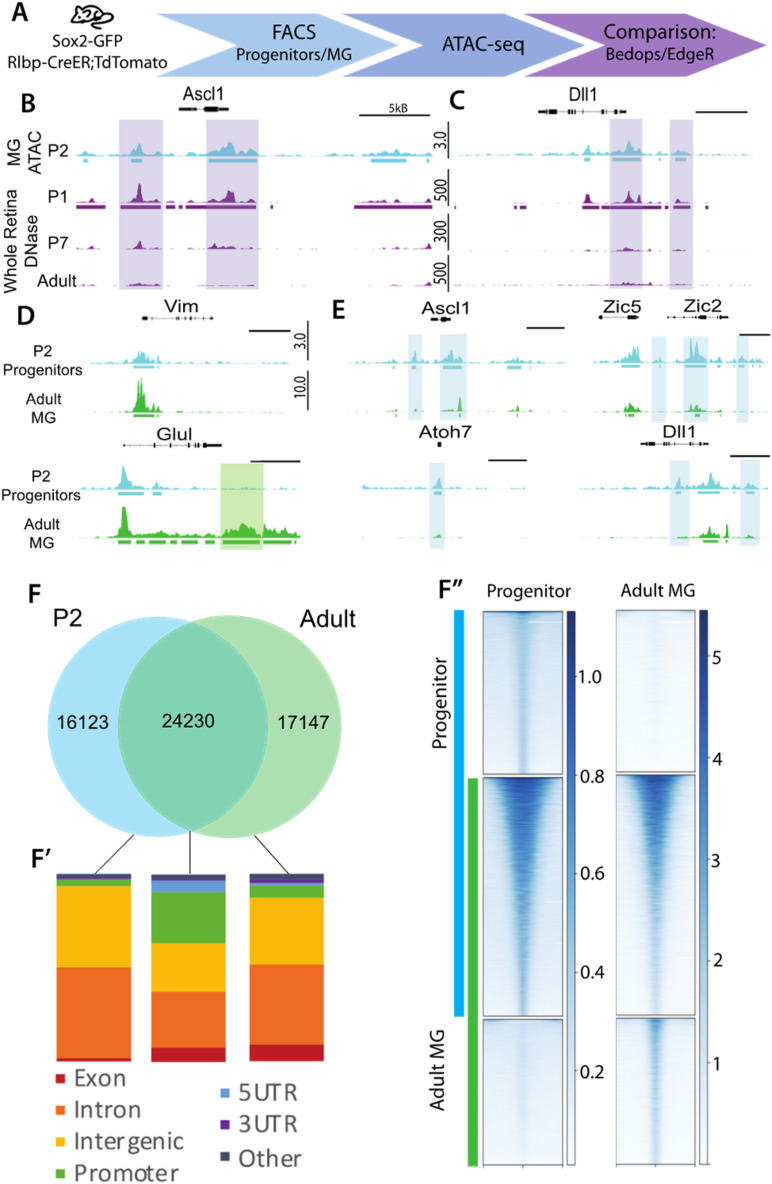
Progenitor and Müller Glial accessibility is generally similar. (**A**) ATAC experimental design. (**B**, **C**). Genomic tracks of P2 progenitor ATAC and whole retina DNase^13^ around Ascl1 and Dll1. Purple bars represent regions of similar accessibility between P1/2 samples. (**D**). Tracks for progenitors and adult MG^9^ ATAC-seq at glial genes and E. progenitor genes. Shaded bars represent regions of interest. Scale bar (**B**–**E**) 5 kb, track heights labeled to the side of tracks (DNase: FKPM, ATAC: reads per million, all heights consistent per sample). F. BEDOPS overlaps of progenitor and adult MG ATAC-seq peak calls. F’. Genomic region annotation of accessibility profiles in F. Annotation values compared via Chi squared test (Shared regions promoter, posthoc p val < 1*10^–250^; intergenic posthoc p val = 1.46*10^–185^[MG], 5.99*10^–37^[Progenitors]; intronic 1.78*10^–196^[MG], 3.84*10^–12^[Progenitors]). F’’. Read density (RPKM) of ATAC-seq in progenitor and adult MG ATAC at accessibility profiles from F.

To determine the differences in the broader epigenomic landscape of retinal progenitors and developing MG, we used Assay for Transposase-Accessible Chromatin (ATAC) sequencing to probe for differences in their accessibility (Fig. 1A). To isolate retinal progenitor cells at P2, we used a knock-in *Sox2*-*Gfp* mouse line that expresses GFP under control of the *Sox2* promoter^10^. At this age, the retina contains a large population of retinal progenitor cells, which are proliferating and producing late-born retinal neurons; these progenitors terminally differentiate into MG between P4 and P5^7,11,12^. The great majority of SOX2 + cells at P2 are retinal progenitors, though there is a small population of SOX2 + amacrine cells that can be distinguished from the progenitors by their high level of GFP (Figure S1). The retinas of P2 pups were dissociated into single cells and the GFP + cells were sorted by Fluorescence-Activated Cell Sorting (FACS); the small number of strongly fluorescent amacrine cells were sorted separately from the more abundant progenitors (Figure S1). To validate that the vast majority of Sox2-GFP + cells were retinal progenitors, we carried out RNAseq and directly compared their transcriptomes with those of retinal progenitors identified from previously published single cell RNAseq (Clarke et al. 2018, Figure S6). The gene expression profiles were highly correlated (Fig S6A). SOX2-GFP + sorted cells were used for two runs of ATAC-seq. Two biological replicates were carried out and we identified approximately 40,000 high confidence peaks that were used for the subsequent analysis. We compared our progenitor ATAC data with DNaseI-seq data from P0, P7 and adult retina, previously generated by our lab^13^. At P0 approximately 30% of the retinal cells are progenitors, while at P7 there are few progenitors remaining at the retinal periphery and none in the adult^11^. Thus, we would anticipate the greatest overlap in accessible peaks between the progenitor ATAC-seq and the P0 retina. Indeed, when comparing P2 progenitor ATAC accessibility to whole P0 retina DNase, the top approximately 40,000 DNase peaks overlapped 73.9%, and when expanding that comparison to the whole DNase set, 92.5% of the P2 ATAC peaks were encompassed by the DNase data. By comparison, only 11.3% of P2 progenitor ATAC peaks overlapped with the top 40,000 Adult retina DNase peaks. We found that progenitor-specific genes, such as *Ascl1*, show similar accessibility in the P0 retina and P2 FACS-purified progenitor cells (highlighted regions, Fig. 1B,C). Comparison of the progenitor ATAC-seq with older P7 and adult whole retina DNaseI-seq showed a reduction in accessibility at regions near progenitor-specific genes, consistent with the loss of progenitor cells as the retina matures.

### Chromatin landscapes in MG and progenitors have a high level of overlap

We compared the progenitor ATAC-seq data to our previously published mature adult MG ATAC-seq to determine if there are specific molecular differences in chromatin accessibility between these two cell types. The accessible chromatin in MG peaks was assessed in both FACS purified MG from adult mouse retina, as well as from MG maintained in dissociated culture. In previous studies, we found that a small amount of rod photoreceptor DNA and RNA is carried along with the adult MG during FACS^9^; however, when we maintain the MG in dissociated culture, very few rods (< 0.1%) survive. Therefore, to reduce the contribution from rod contamination in our downstream analyses, MG-specific regions of accessibility were chosen that overlapped with DNase-seq from cultured P12 MG by BEDOPS to identify peaks that are common to both populations^8^. When we examine the peaks that are present in both the freshly isolated MG ATAC-seq and the peaks from the cultured MG, we find they overlap 93.5% (Figure S2). The approximately 2.7 k peaks that are unique to the adult MG accessible regions are specifically enriched for rod gene-associated accessible domains such as *Crx, Rcvrn,* and *Rho* (Figure S2E), and are likely due to the small amount of rod photoreceptors that contaminate the FACS purified MG.

When we then compared the MG accessible peaks with those of the progenitor cells, we find that there is extensive overlap in overall accessibility. Approximately sixty percent of the progenitor peaks (24,230 peaks) were shared between progenitors and MG, while P2 progenitors had 16 k exclusive peaks, and the adult MG have 17 k unique peaks (Fig. 1F). Analysis of the peaks that are unique to either progenitor cells or MG allowed some general conclusions: (1) Both progenitor cells and MG have similar regions of accessible chromatin near genes that are expressed at high levels in MG; however, the MG show a greater signal at many of these peaks, and also had unique peaks near glial genes that were not present in the progenitor cells (Fig. 1D, highlighted region shows increased signal, Figure S4A). (2) Some genes important for progenitor cell function, but with low MG expression (eg. *Ascl1, Dll1*) had many of the same accessible regions near promoters in both the MG and the progenitors (Fig. 1E), though in many cases the P2 progenitors had more regions of accessibility at these genes than the mature MG (highlighted regions, Fig. 1E). The difference in accessibility between these cell types varies from reduced peak height to a complete loss of some peaks. (3) Promoter regions were over-represented in regions of shared accessibility, while peaks that were unique to either MG or progenitor cells were predominantly found in intronic and intergenic regions (Fig. 1F;F’). Read density in these categories of accessibility showed that shared accessible regions had an overall higher read density in broader regions (p value < 2.2*10^–16^), whereas cell type specific accessible regions had narrow regions of read density (Fig. 1F’’). These differences suggest that while promoter and major regulatory regions retain similar accessibility, many putative regulatory regions differed in accessibility between these cell types, likely reflecting the difference in their respective patterns of gene expression.

### Cis-regulatory binding sites characterize glial development

In order to explore putative regulatory regions that are specific to progenitor and MG populations, we analyzed and annotated regions of changing accessibility. We defined regions of changing accessibility in two ways. First, we identified unique peaks between P2 and Adult MG by BEDOPS^14^, which identifies peaks by overlap on the genome, regardless of size. Alternatively, we analyzed differences in tag density by a read density logFC (fold change) > 2 at all peak locations and selected the top 1,000 regions of Differential Accessibility (DA) for further analysis. These two different pipelines gave similar results for gene ontology and binding motif annotation (Tables S1, S2).

**Figure 2:**
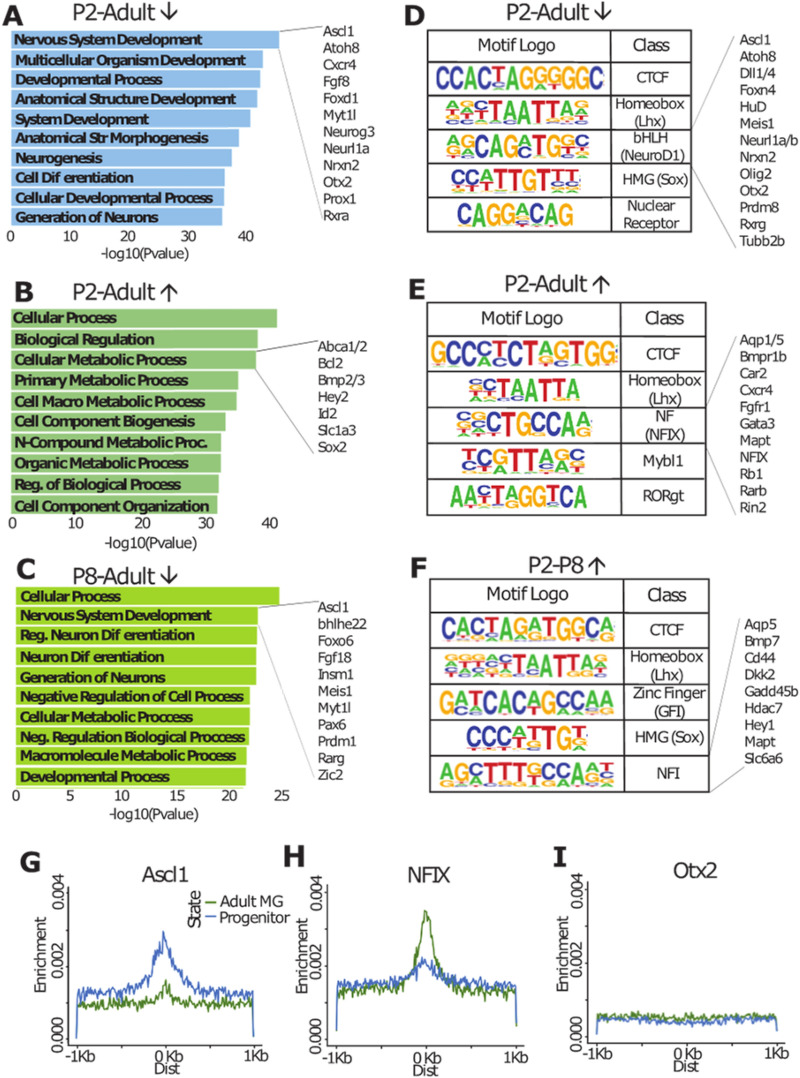
Cis-regulatory binding motifs differ in Müller glia development. (**A**–**C**) Gene ontology associated with accessibility changes (**A**. LOA from P2 to adult, **B**. GOA from P2 to adult, **C**. LOA from P8 to adult). Genes of interest for key ontology categories shown at the right. (**D**–**F**) Predicted motif enrichment for accessibility changes (**D**. LOA from P2 to adult [BEDOPS], **E**. GOA from P2 to adult [BEDOPS], **F**. GOA from P2 to P8 [DA]). Genes of interest for key motifs shown at right. (**G**–**I**). Central enrichment of motifs for progenitor- and adult MG-specific accessibility regions.

When we carried out gene ontology (GO) analysis for those regions that had greater accessibility in the progenitor cells than in the MG (Loss of Accessibility or LOA), we found these regions were associated with genes that were enriched for GO terms of Neural Development/Neurogenesis and Developmental Process/Cell Differentiation (Fig. 2A). The genes associated with the top GO enriched term, Neurogenesis, include known regulators of neural development, including *Myt1l, Neurog3, Otx2, Prox1*, etc. By contrast, the peaks that were not accessible in retinal progenitors, but present in mature MG (ie. Gain of Accessibility or GOA), were associated with genes that were enriched for GO terms of more general cell function: e.g. metabolic and cell process genes (Fig. 2B). Genes associated with these GO terms include those known to be important for glial development and function, including *Hey2, Id2, Slc1a3* and *Bmp* family genes. Those accessible regions present in both cell types are also enriched primarily in metabolic genes (Table S1).

We next performed ATAC-seq on FACS-purified immature P8 MG from *Rlbp1*-*CreER;flox-stop-TdTomato* mice to better understand the dynamic changes in the accessible chromatin that occur as MG mature (Figure S3). MG from P8 mice were used since this age is a crucial stage in MG development^15,16^. The reporter mice and FACS protocol were similar to those described previously^17^. For the P8 sample, there were 49.6 M reads and 16 k peaks by Homer findPeaks, of which 98.35% overlapped with the P7 DNaseI whole retina dataset. We carried out DA analysis as described above between the P8 and adult MG, and the progenitor cells and P8 MG in order to demonstrate changes in accessibility enrichment as progenitors develop to immature MG and as immature MG mature. Interestingly, we found GO terms of Nervous System Development and Generation of Neurons as some of the top Biological Process terms associated with the P8 MG accessible regions that are lost or reduced in the mature MG cells (Fig. 2C) and these are associated with genes important for retinal development, such as *Insm1, Zic2, Meis1,* and *Prdm1,* as shown in the changes from P2 to adult MG. Thus, it appears that many of the putative cis-regulatory regions near genes associated with neurogenesis that are accessible in progenitors are still accessible in the immature P8 MG, and suggests these cells may be more amenable to reprogramming than mature MG.

The peaks of accessible chromatin near genes associated with neurogenesis that are specific to progenitor cells (i.e. not present or reduced in the mature MG) may be relevant to the differences between these cells in their ability to generate neurons. We analyzed these putative neurogenesis-related cis-regulatory elements for enrichment of transcription factor binding motifs using Homer findMotifsGenome against randomly selected background regions. We found that the top five transcription factor motifs enriched in accessible domains specific to progenitor cells at P2 (LOA) were CTCF, LHX (eg. LHX2), bHLH (eg. NEUROD1), HMG (eg. SOX2) and nuclear receptor (Fig. 2D). Genes in these transcription factor families are known to be important for retinal neurogenesis. In particular, the proneural bHLH factors NEUROG2, OLIG 2 and ASCL1 are all expressed in retinal progenitors at this stage^18^. Analysis of the genes associated with the regions enriched for E-box motifs include genes known to be important in retinal neurogenesis, including FOXN4, DLL1, OLIG2, OTX2 and Rxrg (Fig. 2D). When comparing E-boxes with specificity to ASCL1 (CAGSTG) and NEUROG2 (CAKATG)^19^, we found that ASCL1-specific E-boxes had far more distinct central enrichment, especially in regions of progenitor-specific accessibility, whereas NEUROG2 E-boxes are centrally depleted (Figure S4B,C). In addition, ASCL1 has been previously characterized to interact with POU family transcription factors to regulate gene expression^20^. In the P0 retinal ASCL1 ChIP-seq, we found POU binding motifs to be the 15th most enriched motif in progenitor-specific enrichment, occurring in 1.47% of assayed sites over 0.69% of randomly selected background sites (Table S2). Factors such as LHX and SOX are known to be expressed in both progenitors and MG, and thus their presence in progenitor-specific accessible domains is likely indicative of restructuring of regulatory regions during development.

While the accessible chromatin regions specific to progenitors were enriched for proneural transcription factor binding sites, the accessible regions present in the MG, but not present in the progenitors (GOA), have a very different set of enriched transcription factor binding motifs. Although CTCF and LHX motifs were still among the top 5, the NFI (eg. NFIX) motif is enriched in MG (Fig. 2E). This motif was also present in the accessible regions more enriched in immature P8 MG than the progenitors, making this an early marker of MG maturation (Fig. 2F). The motifs enriched in the MG-specific accessible chromatin regions may well reflect the importance of LHX and NFI transcription factors in MG maturation ^21–24^, though NFI factors have also been implicated downstream of neurogenesis genes such as *Pax6*^25^. To help confirm the role of NFI based on motif presence in adult MG-specific accessible domains, we compared genes neighboring NFI binding motifs to known genes regulated by NFI in the process of stem cell quiescence (Fig. 2E,F)^26^. The genes shown to the right of the NFI motifs in Fig. 2E,F are examples of genes present in both datasets. The presence of NFI binding motifs throughout MG accessible domains implicates a complex role for NFI transcription factors in directing the development and maturation of MG cell fate within the retina.

Central enrichment modeling also highlighted the differences between progenitors and MG. For example, progenitor-specific accessible regions were centrally enriched for Homer’s predicted ASCL1 motifs (CAGVTG), with much lower accessibility in the adult MG at these sites (Fig. 2G). The opposite is true for the NFIX motif (CTFCCA): adult MG-specific accessibility profile showed much higher enrichment for NFIX binding motifs within accessible domains than the progenitors (Fig. 2H). By contrast, homeobox domains not enriched in any of these regions (e.g. OTX2; TAATCCY) showed no central enrichment for either progenitor- or MG-specific accessible domains (Fig. 2I). These motif enrichments further support a role bHLH factors in maintaining the neurogenic potential in progenitor cells, and of NFI transcription factors in regulating MG fate.

Since many DNA-binding transcription factors may act in tandem with other transcription factors in a combinatorial manner to regulate gene expression, we analyzed co-occurrence between the top predicted binding motifs for progenitor (Figure S4C) and adult MG (Figure S4D) accessible domains. Through this analysis, we found that SP1 and E2F domains commonly co-occur in both progenitor- and MG-specific accessible regions. We also found some co-occurrence between BRN1, HOXB4, FOXP1, and LHX2 motifs, particularly in progenitor-specific accessible domains. Overall, co-occurrence analysis does not indicate any particular co-regulatory group that might interact with the major motifs that are associated with progenitor (ASCL1) or glial (NFIX) cell fate.

### Ascl1 overexpressed in MG binds to potential Cis-regulatory regions near neurogenic genes

**Figure 3:**
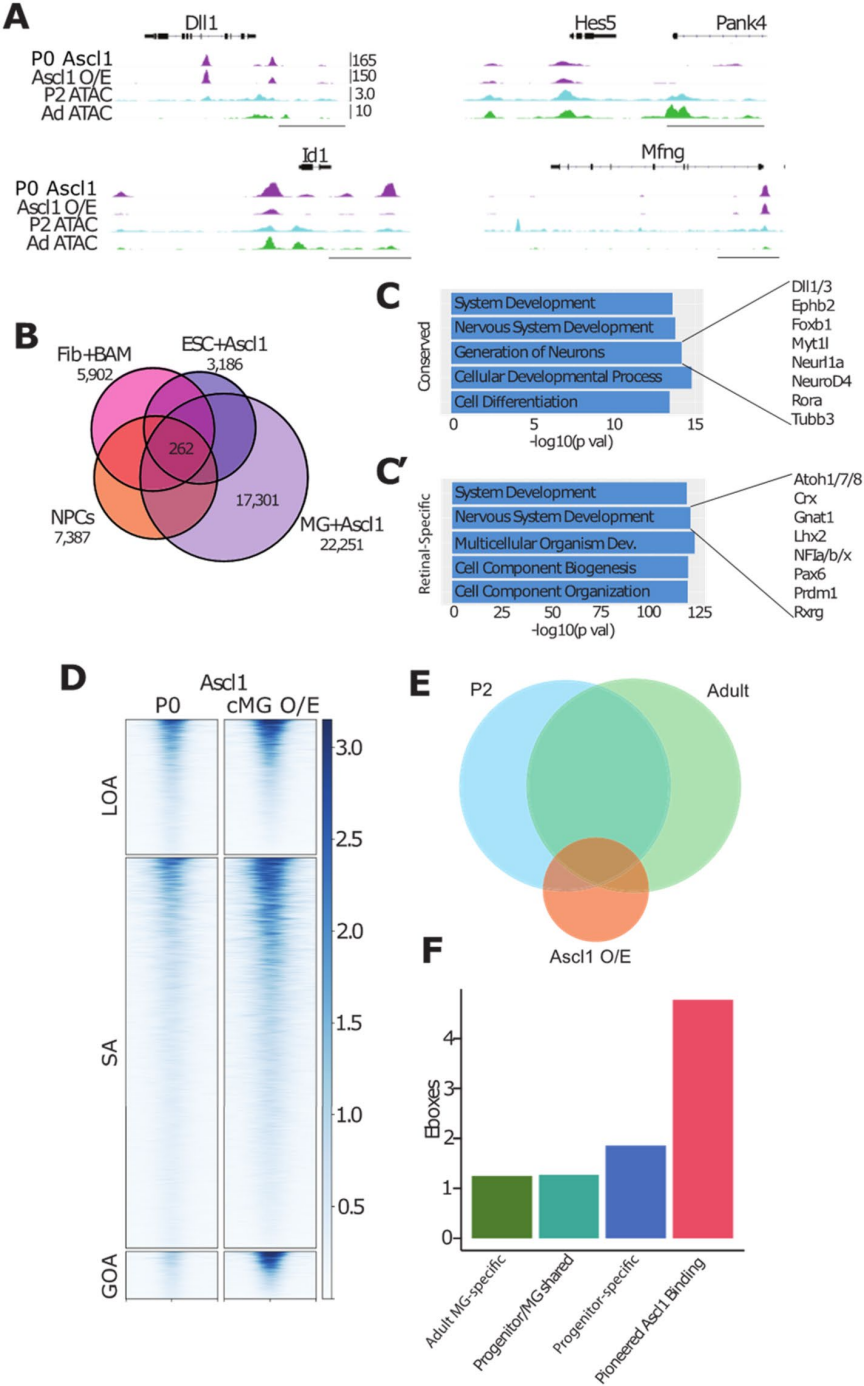
*Ascl1* Overexpression in Culture Opens a Retinal Neurogenic Niche. (**A**) Genomic tracks of ASCL1 ChIP for developmental (P0) ASCL1 binding and overexpression in cultured MG (O/E), and ATAC for P2 progenitors and Adult MG around developmental targets of ASCL1. Track heights for all (FKPM for ASCL1 ChIP, reads per million for ATAC) labeled in top left track. Scale bar for 5 kb at lower right on all tracks. (**B**) Overlaps between ASCL1 ChIP from cultured MG and published ASCL1 ChIP loci: Neural Progenitor Cells (NPCs), Fibroblasts + BAM (BRN2, ASCL1, MYT1L)^28^, and Embryonic Stem Cells (ESCs) + ASCL1^29^. Total peak numbers for each dataset, as well peaks shared between all datasets, and those only found in the retinal dataset. (**C**) Gene ontology by GProfiler for intersection of all datasets and retinal-only peaks. Genes of interest for neurogenic ontology categories at right. (**D**) Heatmap of ASCL1 ChIP-seq read density across ATAC accessible regions (LOA top, shared accessibility [SA] middle, GOA bottom). Left heatmap demonstrates normalized ChIP reads for ASCL1 in P0 retinas (P0), while right demonstrates normalized reads for ASCL1 overexpressed in cultured MG (ASCL1-OE) + /– 1 kb from peak center. (**E**) Representation of ATAC overlaps with ASCL1 ChIP peaks. F. Average numbers of ASCL1-related E-boxes predicted in each of the ASCL1-binding overlap categories represented in the ASCL1 bubble in (**E**).

To better understand the role of ASCL1 in progenitors, and how it alters the epigenome in MG during reprogramming, we compared ASCL1 ChIPseq in newborn mouse retinal progenitors with that of *Ascl1*-reprogrammed MG. Obtaining sufficient glial cells for ChIP-seq after reprogramming in vivo has not yet been possible, due to the large number of cells required for ChIP-seq and the relatively small numbers of reprogrammed cells present in the retina after the in vivo reprogramming protocol. However, since *Ascl1* is sufficient to reprogram MG to a neurogenic state in vitro that is very similar to what we observe in vivo^27^, we compared ASCL1 ChIP-seq in newborn mouse retinal progenitors with that of MG that had been isolated from P12 mice and expanded in vitro. In both progenitors and reprogrammed MG, ASCL1 binds near developmental genes such as Dll1, Hes5, Id1, and Mfng (Fig. 3A). This binding is consistent with accessible domains in progenitors or adult MG (Fig. 3A). We compared our ASCL1 ChIP-seq data to previously published ASCL1 ChIP data in neural progenitor cells (NPCs, Wapinski et al. 2013), as well as ASCL1 overexpressed in embryonic stem cells (ESCs, Casey et al. 2018), and in fibroblasts with additional factors BRN2 and MYT1L (Fib + BAM, Wapinski et al. 2013). There were 262 regions in common between all published datasets and *Ascl1* overexpressed in cultured MG, and 17,301 binding sites that were only found in our retinal dataset between those compared (Fig. 3B). We found similar overlaps with developmental ASCL1 binding, with 287 shared ASCL1 binding sites (Figure S5). The shared regions of ASCL1 binding were associated with neuronal gene ontology categories Nervous System Development and Generation of Neurons around genes such as *Myt1l*, *NeuroD4,* and *Tubb3* (Fig. 3C). ASCL1 binding found only in MG reprogrammed with ASCL1 was similarly associated with the Nervous System Development gene ontology category around retinal development-related genes such as *Atoh7, Crx,* and *Pax6* (Fig. 3C’).

A comparison of the ASCL1-ChIPseq with at ATAC-seq accessible domains in progenitors and adult MG shows that ASCL1 binds to progenitor-specific regions, as well as regions shared between progenitors and adult MG, with approximately 12.5 k shared peaks (Fig. 3D). Progenitor ASCL1 binding overlaps with progenitor accessibility by 13,151 peaks (Figure S5D). In progenitor accessible domains that are also bound by overexpressed ASCL1 in MG, motif analysis demonstrates strong enrichment in bHLH E-box binding motifs, but additionally POU binding motifs become further enriched, found in 10.66% of assayed regions over 7.99% in background sequences (Figure S5E; Table S2). When ASCL1 is over-expressed in MG, it binds to adult MG-specific accessible domains (Fig. 3D). In addition, ASCL1 binds to a strong distinct niche in adult MG: regions neither natively open in the glia nor developmentally appropriate (Fig. 3E). We call these pioneered ectopic sites.

Casey et al. 2018 recently demonstrated that ES cell reprogramming with ASCL1 revealed a particular class of accessible regions with repeated E-box motifs^29^. Similar to that study, we find that those regions of the DNA that are inaccessible in MG or progenitors, but bind ASCL1 when over-expressed (pioneered sites) had on average 4.7 E-boxes per peak, while other ASCL1-bound sites in MG have fewer than 2 E-boxes on average (Fig. 3F). This result is consistent with the prior result that demonstrated that the pioneering activity of ASCL1 is dependent on E-box repeats around new binding sites, though developmentally appropriate binding sites do not contain these repeats and can bind ASCL1 regardless of current accessibility.

### Immature MG expression is intermediate to progenitors and mature MG

**Figure 4:**
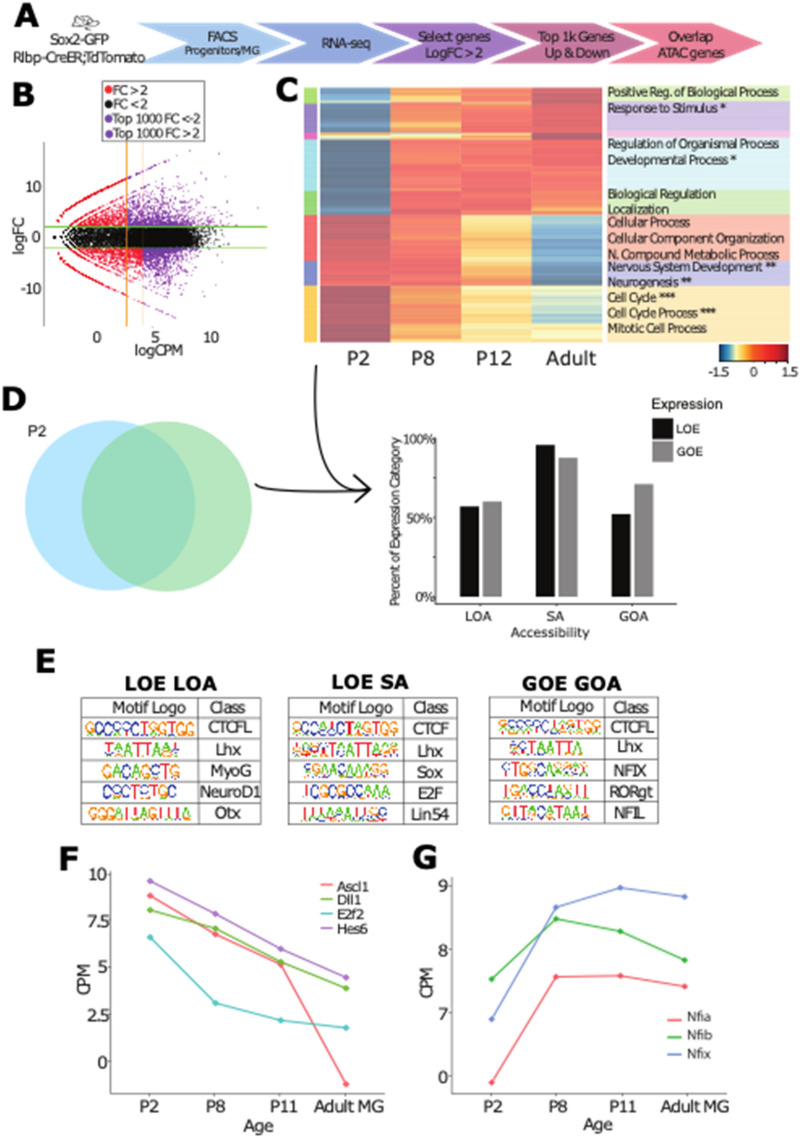
Glial expression and accessibility profiles follow developmental trends. (**A**) Experimental design for FACS isolation and RNA-seq of developing MG. (**B**) MA plot of expression from progenitors to adult MG demonstrating thresholds for filtering high fold change and counts for the top genes with changing expression. (**C**) Euclidian hierarchical clustering by expression changes from P2 to Adult, heat map of top gene expression (expression scale lower right), and related gene ontology categories. Categories marked with a *indicates categories that overlap with ATAC MG increased accessibility. Those marked with a** indicate where progenitor-specific accessibility overlaps, and ***indicates where shared accessibility overlaps. (**D**) Top gene expression changes were cross-compared with ATAC profiles. The percentage of genes that increase or decrease expression are represented on the y axis, split by ATAC categories (on the x axis). (**E**). Motif enrichment for accessible regions with gained expression and accessibility (GOE GOA), lost expression and accessibility (LOE LOA), and lost expression and shared accessibility (LOE SA). (**F**, **G**) Expression profiles of (**F**) progenitor genes of interest, and (**G**) NFI transcription factors.

To better understand the molecular basis for the difference in accessibility between the progenitors and the MG, we carried out RNA-seq on FACS purified progenitors and immature and mature MG (Fig. 4A). Progenitors were sorted using P2 *Sox2-Gfp* mice as previously described. MG from P8, P11, and Adult retinas were sorted using *Rlbp-creER:flox-stop-tdTomato* reporter mice to sample stages of MG maturation. Differential expression analysis was performed to demonstrate overall differences between progenitors and mature MG. We selected the 1,000 most highly expressed genes with logFC > 2, which were differentially expressed in MG (Gain of Expression, GOE) or in progenitors (Loss of Expression, LOE) by logCPM (counts per million) values (Fig. 4B).

Genes were clustered via k-means clustering, and GO terms were associated with specific clusters (Fig. 4C, Tables S3, S4, S5). Genes that showed marked increases from P2 progenitors to adult MG had somewhat general Biological Process terms (“Response to Stimulus”, and “Biological Regulation”), while genes that were highly expressed in progenitors, but expressed at much lower levels in MG, were associated with more specific GO terms that reflect the unique functions of the progenitor cells: “Cell Cycle,” “Nervous System Development” and “Neurogenesis” (Fig. 4C, Table S5). We compared our data with the single cell RNA sequencing (scRNAseq) data from Clark et al. 2019. Fig S6A shows that gene expression in FACS sorted progenitor cells at P2 correlates highly with that of progenitor cells from Clark et al. 2019. Additionally, we compared the top 1,000 markers of P2 RPCs from our data with markers of P2 progenitors identified from scRNAseq. To determine the top markers from P2 progenitors in single cell RNA sequencing data, the subset of cells that were identified as either glia or progenitors (defined by the cluster of cells expressing *Slc1a3* and *Rlbp1* most highly expressed in both P2 and P14 data) were used for comparison using Seurat’s findmarkers function. Only genes with an adjusted p value of < 0.05 were considered. Fig S6B shows markers of progenitors shared in both datasets. Many of these markers have been previously described in progenitor cells, including genes related to the mitotic cell cycle and neurogenesis (Fig. S6 C-E).

We also compared the gene expression in the immature MG at P8 and P11. These immature MG expressed many of the genes that are expressed in the retinal progenitors, albeit at a lower level than the progenitors, but in addition they expressed genes more characteristic of mature MG (Fig. 4C). The heatmap shows that four of the five clusters of "MG-specific" genes had the greatest increase between P2 and P8. Genes that lost expression most rapidly, in the tan cluster are primarily associated with the cell cycle. In contrast, the cluster (blue) most closely associated with the GO terms of “Neurogenesis” and “Nervous System Development” declined over the first postnatal week more gradually, but had very low levels of expression in mature MG (Fig. 4C). Comparing the changes in gene expression during MG maturation revealed an intermediate level of expression in young MG, with progenitor gene expression retained at relatively high levels through P8.

Are the changes in gene expression between MG and retinal progenitors reflected in their chromatin accessibility? To answer this question, we compared the genes that change in their expression (GOE, LOE) with the developmental ATAC categories (GOA, SA [Shared Accessibility] LOA). To make this direct comparison, we annotated the ATAC peaks to neighboring genes using GREAT. These annotated gene lists were then overlapped with GOE and LOE gene lists, yielding a percent of total genes for GOE and LOE categories for each accessibility profile (Fig. 4D, Table S6). These overlaps demonstrate areas where expression and accessibility are both positively and negatively correlated, potentially indicating regions of positive and negative expression regulation. Accessible chromatin regions that were shared between progenitors and MG were especially highly associated with genes both in the increasing and decreasing gene expression (GOE and LOE) categories (Fig. 4D). In an effort to focus on areas of positive gene regulation, we specifically looked at areas where nearby accessibility changes were positively correlated with gene expression changes. The regions of lost expression and accessibility (LOE/LOA) are best associated with Neurogenic GO categories, and this trend is continued in comparisons from P8 to Adult MG LOE/LOA (Table S7).

By overlapping the ATAC-seq data with the gene expression results, we were able to better define some of the differences in transcription factor expression and putative binding at gene-associated regions that might be positively regulating the difference in neurogenic competence between these two cell types. For example, LOE/LOA regions were enriched for binding motifs for proneural bHLH factors, whereas those genes that gain accessibility and expression (GOE/GOA) were enriched for binding motifs for NFI and ROR (Fig. 4E). These results are in line with those obtained from the analysis of the ATAC seq results (described above), indicating that the binding motifs that differed between these cells were associated with those genes that similarly changed in expression. Transcription factors that bind to these motifs were also found to change expression in accordance with changes in motif accessibility (Fig. 4F,G). Interestingly, genes with accessible peaks in both MG and progenitors (shared accessibility), which lost expression in mature MG (LOE/SA) are associated with the mitotic cell cycle (HyperGTest, p-value = 5.89 × 10^–23^). These regions were enriched for the binding motif for the cell-cycle regulator E2F transcription factor (Fig. 4D,E). These observations suggest that the differences between progenitor cells and MG in their cell proliferation may not be regulated by changes in chromatin accessibility.

### Ascl1 is sufficient to induce neurogenesis in immature MG

Our previous results showed that the chromatin landscape may play a role in limiting the competence of MG to regenerate neurons: HDAC inhibition is necessary for ASCL1 to reprogram adult MG to neurogenic progenitors, but this inhibition is not necessary in younger MG^8,9^. Given the increased chromatin accessibility at progenitor genes of immature MG at P8 (compared with mature MG), we predicted that P8 MG might be more easily reprogrammed to a neurogenic state. To test the hypothesis, we overexpressed *Ascl1* in retinal progenitors and MG at various times during postnatal development. In order to trace the lineages of retinal progenitors and MG, we used a tamoxifen-inducible creER mouse driven by one of two promoters to activate a fluorescent reporter. For control mice, we used *Glast-CreER:flox-stop-*

**Figure 5:**
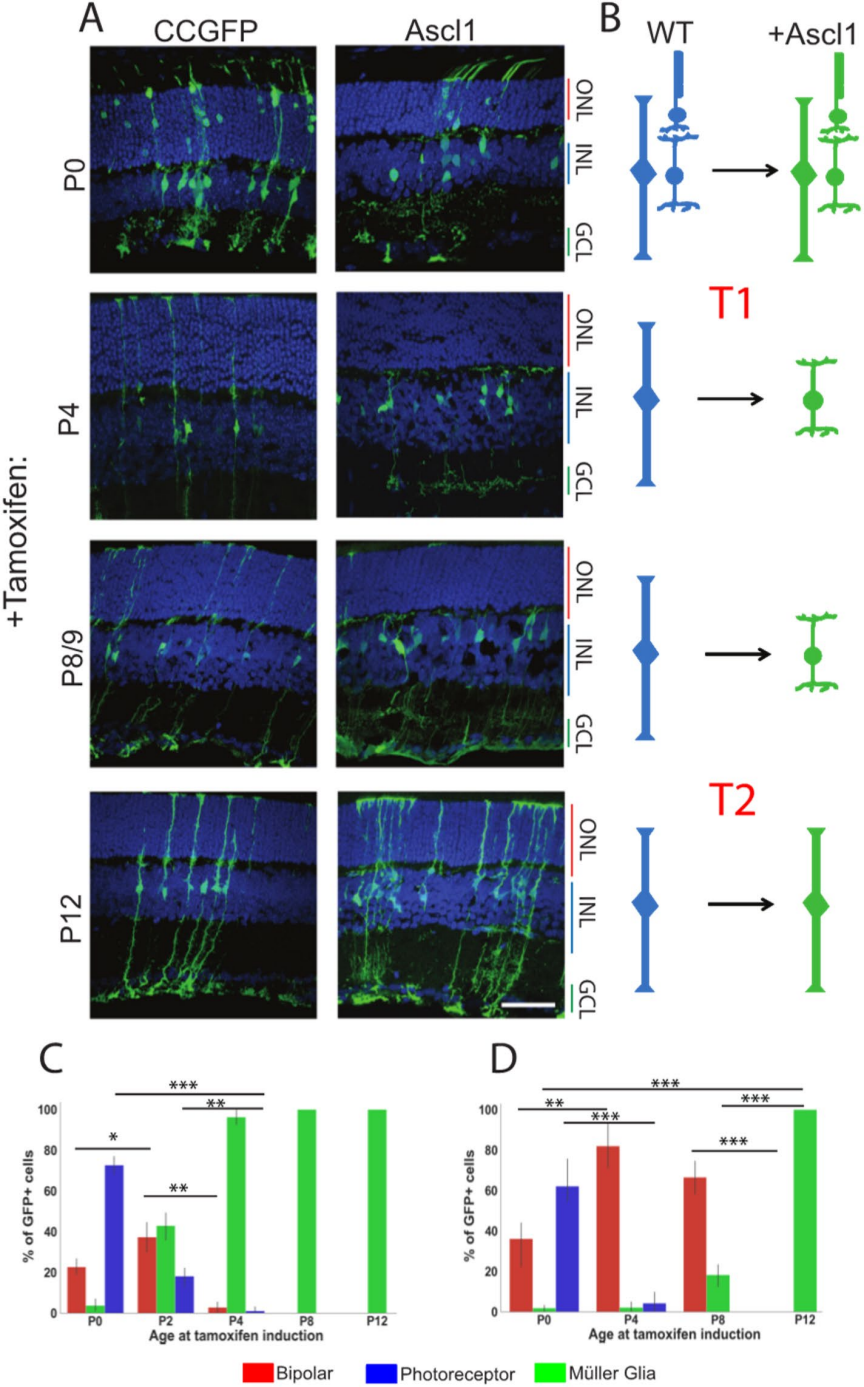
*Ascl1* overexpression at young ages is sufficient to generate bipolar neurons. (**A**) Cell tracing of progenitors and MG was induced with *CC-GFP* or *Ascl1-ires-GFP* on a *Glast* promoter at P0, P4, P8/9, and P12. All analyzed at P21. Scale bar 50 um. (**B**) Graphical summary of reprogramming observed with Ascl1. (**C**) Cell counting quantification of GFP cell tracing in (**A**). (**D**) Cell counting quantification of Ascl1 induction tracing in (**A**).

*CC-GFP*; mice received an intraperitoneal injection of tamoxifen to initiate the recombination at P0, P4, P8, or P12, and were collected at P21. Reporter expression was observable within 24 h of tamoxifen injection in P8 mice (Figure S7). Cell identities were classified by cell body localization, morphology, and staining for OTX2 and SOX9. The promoters are glial-specific in adult mice, but are expressed in retinal progenitors at P0. When cells were lineage traced at P0, the late-born types of neurons are present in the progeny: 72.6% of GFP + cells had a photoreceptor fate, 22.7% were bipolar cells, and the remaining cells were MG (Fig. 5A–C). These are similar to the ratios found when these cells that are birth-dated at this age in mice^11^. Lineage tracing at P2 leads to 18% rods, 37% bipolar cells, and the remaining were MG (Fig. 5C). By P4, the percentage of GFP + neurons was significantly reduced with only 2.72% of cells having a bipolar cell fate, while the remainder throughout the retina were MG (Fig. 5B). At the far retinal periphery however, some retinal progenitor cells were still present at P4, and so some rods and bipolar cells were observed in this area (Figure S7 C). These results demonstrate that murine retinas retain multipotent progenitor cells for the first 3–4 postnatal days. In mice older than P4, 100% of GFP + cells were MG in central retinal regions, though some neurons were present in the lineage in peripheral retina. From P8 and on, 100% of GFP + cells were MG throughout the retina.

To determine whether immature MG can be reprogrammed to a neurogenic state with Ascl1 alone, we drove expression of Ascl1 at the same ages that we had used to trace WT cells. We used a previously described system for driving Ascl1 in MG: *Glast-CreER;Flox-stop-LNL-tTA;TetOCMV-Ascl1-ires-GFP*^8,9^. With the induction of *Ascl1* expression at P0, while there appears to be a trend in increasing bipolar cells, ratios of GFP + neurons were not statistically significantly different from WT lineages. This suggests that the level of *Ascl1* is expressed at sufficient levels in retinal progenitors to sustain neurogenesis, and that additional *Ascl1* has little effect. At P4 and P8, however, over-expression of *Ascl1* in immature MG has a dramatic effect on the cells: 75% and 65% of GFP + cells, respectively, are now bipolar neurons, with few—if any—photoreceptor neurons. When we delay induction of *Ascl1* to P12, however, 100% of GFP + cells are now MG, and at this age, expression of *Ascl1* alone is no longer sufficient to reprogram the MG to neurons, similar to results previously reported (Fig. 5A,D)^8^. These results show there is a rapid change in the competence of cells to generate rods with *Ascl1*-overexpression summarized in Fig. 4B. Some major change (Transition [T]1) occurs at P4 in the progenitor cells as they become MG to restrict the ability of ASCL1 to generate rods. A second major change (Transition [T]2) appears to control the ability of MG to generate bipolar cells, and this occurs after P8. After this time, damage to the retina and inhibition of histone deacetylases is needed to induce neurogenesis from the MG.

### Retinal neuron chromatin overlaps similarly with progenitor and MG

**Figure 6:**
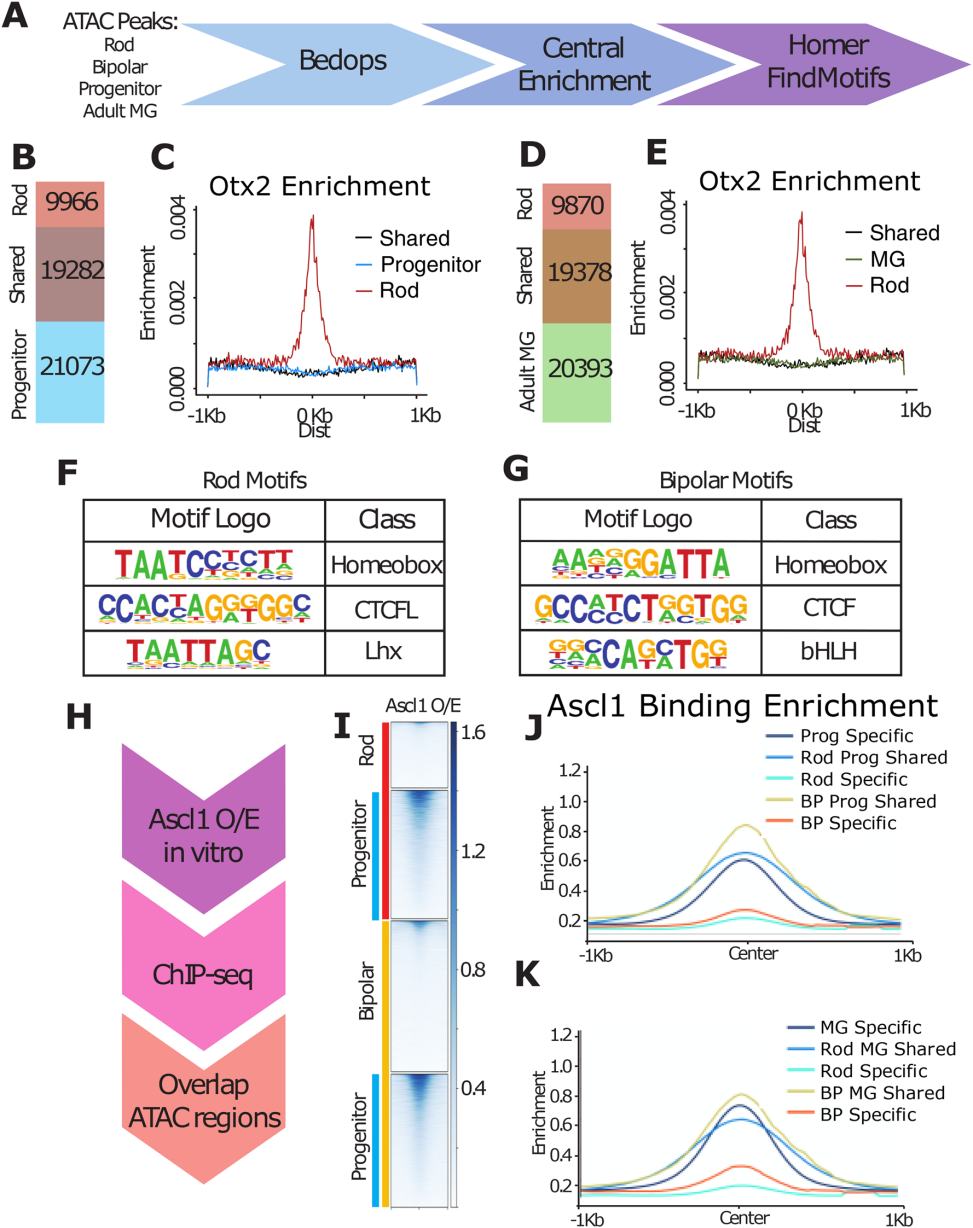
Ascl1 binding to accessible regions of rods and bipolar cells. (**A**) Analytical design comparing the accessible regions in neurons^9,30^ (ATAC-seq from progenitors and MG). (**B**, **D**) Rod chromatin accessibility overlaps with both progenitor (**B**) and adult MG (**D**) accessibility by 19 k regions. (**C**, **E**) OTX2 predicted central enrichment is high in Rod-specific accessible regions, and low in regions shared with progenitors (**C**) and adult MG (**E**). (**F**, **G**) Predicted motifs for rod-specific (**F**) and Bipolar-specific (**G**) accessible regions. (**H**) Experimental design for ASCL1 ChIP-seq. (**I**) ASCL1 ChIP-seq read density (RPKM) in Rod, Bipolar, and Progenitor accessible regions. (**J**, **K**) Central enrichment (RPKM) for ASCL1 binding in rod, bipolar, and progenitor (**J**) or adult MG (**K**) accessible regions.

The changes in neurogenic competence that occur as cells transition from progenitor cells to MG even with forced *Ascl1* expression might be due to changes in their epigenome. To determine if DNA accessibility might underlie these differences in (1) neurogenic competence, and (2) fate restriction to predominantly bipolar cell neurogenesis, we compared ATAC-seq data from progenitor cells, MG, rod photoreceptors^30^, and bipolar cells^9^ (Fig. 6A). We found that both progenitor cells and adult MG accessible regions overlapped with rods by approximately 19 k peaks or 47.5% of glial/progenitor-accessible domains (Fig. 6B,D). A similar analysis of bipolar cells and retinal progenitors or MG gave very similar results: bipolar cell accessibility overlapped with progenitor accessible regions by approximately 20 k peaks (50% of progenitor domains), and with MG accessible regions by approximately 17 k peaks (42.7% of glial domains) (Figure S7). For all overlaps, GO categories were similar between comparisons to progenitors or MG (Table S8). As OTX2 is important in the generation of both cell types^31^, we looked for central enrichment of this motif (TAATCCY) in both neuronal-specific and MG-shared accessible regions. For rods, the rod-specific accessible regions (ie. not present in the MG or the progenitor cells) are strongly centrally-enriched for OTX2 binding sites (Fig. 6C,E). We did not see a similar central enrichment for OTX2 in the accessible regions shared between rods and progenitor cells or rods and MG, or in those regions specific to progenitors or MG. A similar analysis of the overlapping and uniquely accessible regions for bipolar cells and either MG or retinal progenitors gave strikingly similar results: the accessible regions that are unique to bipolar cells and not shared with progenitor cells or MG show central enrichment for the OTX2 binding motif; but, regions shared among these cell types did not have this signature (Figure S8). Thus, the ability of ASCL1 to promote the bipolar fate is not due to MG having an epigenome that is more similar to bipolar cells than rod photoreceptor cells in OTX2 accessible sites.

Overall motif enrichment in rod and bipolar neurons was additionally quite similar. Rod-specific regions—when comparing to either MG or progenitors—were enriched for homeobox and CTCF domains (Fig. 6F, Table S2). Shared accessible regions in both comparisons showed enrichment for CTCF, KLF, and ETS binding motifs. Bipolar cell accessible regions that were shared with both progenitor cells and MG also showed enrichment for these TF binding motifs. Interestingly, in both comparisons, bipolar cell-specific accessibility demonstrated enrichment not only for homeobox domains, but also for proneural bHLH domains (Fig. 6G). The presence of bHLH accessibility in bipolar cells may underlie the fact that MG primarily generate bipolar cells with the overexpression of *Ascl1*.

### ASCL1 binding sites are enriched in bipolar-specific accessible regions

These results imply that bHLH transcription factors such as ASCL1 may play a specific role in directing MG to a bipolar cell fate. We next looked at predicted central enrichment of ASCL1 in these neuronal populations. Rod-specific accessible regions enriched over progenitors or adult MG demonstrated some central enrichment of predicted ASCL1 binding motifs, though the overall enrichment did not appear to be notably elevated from regions with accessibility shared with progenitors or MG. By contrast, bipolar cell accessible regions that are specifically enriched over progenitors or adult MG had distinct central enrichment of predicted ASCL1 binding sites, which is also found in shared accessible regions, though to a lesser extent (Figure S8).

In addition to this predictive analysis, we compared neuronal accessibility to Ascl1 binding when overexpressed in cultured MG (Fig. 6H). Regions predicted to be enriched in ASCL1 binding in retinal neurons demonstrated that Ascl1 also binds more strongly to bipolar accessible domains as compared to rod accessible domains (Fig. 6I). Ascl1 binding was highest for progenitor and glial-shared accessible regions, but there was distinct central enrichment for ASCL1 binding in bipolar-specific accessible domains (Fig. 6J,K, Figure S9). Binding of ASCL1 ectopically demonstrates that expression of this TF in MG may further direct fate decisions more towards bipolar interneurons than photoreceptor neurons.

## Discussion

In this study, we have explored the epigenetic profile of retinal progenitors and MG alongside their gene expression profile as these cells change in their neurogenic potential. We have demonstrated epigenetic evidence of putative cis-regulatory elements that change in accessibility through development and potentially regulate the maturation and neurogenic potential of the MG. Furthermore, we have shown that the intermediate profile of immature MG allows for improved reprogramming to bipolar neurons, thus revealing transition states in the neurogenic potential of immature mammalian glia.

In order to explore the potential mechanisms for regulation of epigenetic regenerative capacity in the mammalian MG, we performed ATAC-seq on postnatal neurogenic retinal progenitors as well as on developing and mature MG. We found that the epigenomic landscape of progenitors and mature MG were very similar, with approximately 60% of progenitor accessible domains shared with mature MG, consistent with similarities in gene expression between progenitors and MG^15,32^. These shared regions include glial-expressed genes as well as progenitor-specific genes and were particularly enriched around promoter regions. Cell-type specific accessible regions were enriched in intronic and intergenic regions of the genome, consistent with previous evidence that enhancers may be more dynamic during development^33^.

Accessible regions that were enriched in the retinal progenitors were specifically associated with developmental and neurogenic genes. This was reinforced by expression analysis, demonstrating that progenitor-specific genes, enriched in GO categories associated with early development and neurogenesis, showed more than 2 logFC compared with MG. Many regions of accessibility that were reduced or lost as the MG mature were associated with these same genes that lost expression during postnatal development, reinforcing that accessibility correlates with expression. Motif enrichment analysis of the progenitor-specific accessible domains revealed specific enrichment in bHLH binding domains. This class of transcription factor is well-characterized as part of the retinal developmental process, though there are many variations of E-box motifs in the genome that are relatively specific to different bHLH domains. Aydin et. al 2019 recently described variations of E-box specificity that contribute to neuronal subtype (CAGSTG for ASCL1 and CAKATG for NEUROG2)^19^; we found a nonspecific E-box enriched by HOMER, consistent with progenitor cell potential to generate multiple types of neurons (both excitatory and inhibitory), with the fourth position in the motif being equally probable to be A or C. However, assaying for specific E-box motifs demonstrates a preference for the ASCL1 motif throughout. The loss seen in bHLH binding motifs in the transition from progenitors to MG reflects the decline in expression of ASCL1 and related bHLH TFs as progenitors transition to MG.

As MG mature, the cells increased in both accessibility and expression associated with genes important for glial function. There was a correlation between increases in nearby accessible chromatin and genes that increased in expression during glial maturation. Young MG in particular had a unique intermediate profile. Glial genes that were highly increased in expression in adult MG gain accessibility before P8, but this is not accompanied by a similar drop in progenitor gene expression.

Cis-regulatory accessibility continued to change and develop during MG maturation. While many enriched motif classes were common between decreasing and increasing accessibility, the NFI binding motif was uniquely enriched in the developing glia. NFI domains were similarly enriched surrounding genes that increase expression and gain accessibility, indicating a putative role in the development and maturation of the glial fate. These NFI domains were enriched in accessible regions even in the younger P8 MG, consistent with evidence for an intermediate profile of immature MG. NFI transcription factors are relevant to the developing glia as they are well known for their role in CNS glial development^24,34,35^. In addition, NFIA/B/X are expressed in retinal progenitors and MG, and conditional knockouts of these transcription factors in the developing retina are associated with defects in gliogenesis and the production of bipolar neurons^36^. NFIB has been associated with neurogenesis as a downstream factor from PAX6^25^, and this increase in enrichment for NFI binding sites could still be associated with multiple fate decisions including neurons. However, this could still direct fate decisions, especially as changes to NFI expression in development predominantly affects the generation of MG and bipolar neurons, and has less of an effect on rods^36^. As progenitors develop, there is an acquired association of NFI motifs with LHX2 binding sites, which may be related to guiding the neurogenic potency of late retinal progenitors^37^.

Though RNA-seq and ATAC data demonstrate epigenomic changes that co-occurred with changes in gene expression, not all gene expression changes were associated with changes in accessibility. This is best demonstrated with cell cycle and proliferation-related genes. These regions are associated with E2F binding motifs, consistent with their roles in regulating mitotic proliferation^38,39^. Expression of genes associated with the mitotic cell cycle declined as progenitors withdrew from the cell cycle. However, regions of accessibility associated with these genes changed little in their accessibility between progenitors and MG. This suggests that the loss of proliferative capacity in maturing MG may not be limited by epigenetic accessibility.

Our results showed that the immature (P8) MG have an epigenome intermediate between the progenitors and adult MG. Since efficient reprogramming of mature MG requires a combination of ASCL1 and HDAC inhibition, we postulated that the P8 MG might be more efficiently reprogrammed to a neuronal fate with Ascl1 alone. We found that induction of Ascl1 alone in immature MG was indeed capable of inducing a neuronal/bipolar cell fate, consistent with our hypothesis that epigenomic changes in accessibility limit mature MG from regeneration. Interestingly, the P8 MG already appeared to be fate restricted with respect to the types of neurons produced from *Ascl1* over-expression, with two distinct transition periods. In newborn mice, lineage tracing and birthdating studies have shown that progenitors generate three types of neurons: rods, bipolar cells and amacrine cells^11^; however, early in MG development (e.g. P4), the MG cells lose their ability to generate photoreceptors and amacrine cells (T1), even with *Ascl1* over-expression. Somewhat later in MG development (eg. P10) as the cells mature, they lose their ability to generate bipolar cells from *Ascl1* over-expression alone, though the addition of HDAC inhibitors and injury can restore their neurogenic potential^8,9^.

Do changes in accessible chromatin account for the bipolar fate restriction that occurs during the T1 transition? To address this question, we compared the open chromatin landscape of bipolar cells and rod photoreceptors to determine the degree of similarity between these neuronal cell types and MG or progenitors. Overall, the shared accessible regions between progenitors and either type of neuron (rod or bipolar cell) were very similar to the shared regions between MG and these types of neurons. Moreover, the newly accessible sites in rods or bipolar cells were not present in either progenitors or MG. Thus, the degree of similarity in accessible chromatin between progenitors or MG, on the one hand, and rods or bipolar cells, on the other, are not sufficient to explain the bias in bipolar cell generation from MG. Nevertheless, in comparing neuron-specific accessible regions from rods and bipolar cells, we found that bipolar cell-specific accessible regions were more highly enriched for bHLH motifs than rod-specific accessible regions. Also, over-expression of *Ascl1* in MG results in ASCL1 binding to more bipolar cell-specific accessible regions than rod-specific regions. Similar enrichment for E-box accessibility in a variety of bipolar cells was also shown by Murphy et al.^40^ and this may help to explain why the overexpression of *Ascl1* in MG preferentially generates bipolar cells in vivo, though the factor is necessary for the generation of both bipolar cells and photoreceptors during development. Our results thus suggest that a reprogramming strategy using *Ascl1* alone will be unlikely to regenerate rod photoreceptors, and additional transcription factors will likely be needed.

In sum, we have characterized the ways in which the accessible chromatin landscape changes as retinal progenitors differentiate into MG. Developing MG lose expression of neurogenic genes and accessibility of related cis-regulatory elements, and gain accessibility of glial-defining NFI binding sites. However, young MG demonstrate intermediate profiles. By P8, MG demonstrate early gains in NFI binding sites, while retaining progenitor-like expression and accessibility. This intermediate profile translates into neurogenic potential: prior to P12 overexpression of *Ascl1* alone is sufficient to induce neurogenesis in MG. The affinity for bipolar neurons appears to be in part due to the preference of ASCL1 for binding to bipolar cell specific accessible regions, and not due to inherent overlaps in MG chromatin accessibility with bipolar cells vs rods. Overall, our results show unique transition states in the development of glial cells that restrict their neurogenic potential, which correlate with changes in the epigenome.

## Methods

All methods were carried out at the University of Washington in accordance with approved guidelines and regulations.

### Mice

All mice were housed at the University of Washington Department of Comparative Medicine. All procedures were carried out using protocols approved by the University of Washington’s Institutional Animal Care and Use Committee. Mice expressed Cre-recombinase under the *Glast* promoter from Jackson Labs with *Rosa-flox-stop-tTA* (Jackson labs) in combination with either *tetO-mAscl1-ires-GFP* (M. Nakafuku University of Cincinnati) or *CCGFP*, These mice have a mixed background of 129/SvJ and C57BL/6 J*.* Mice having *EGFP* knocked-into the *Sox2* open reading frame were obtained from Jackson Laboratories (Stock: 017,592) and bred to generate P2 litters^10^. *Rlbp1CreERT2* mice were crossed to *R26*-*stop*-*flox*-*CAG-tdTomato* mice (Jackson Labs, also known as Ai14; 129SvJ background). Mice of both sexes were used for this study and analyzed together in their respective treatment groups. Mouse and cell numbers utilized are catalogued by experiment in Table S9.

### Previously published data

SRA accession numbers for all referenced datasets used in this study are listen in Table S10.

### Injections

Intraperitoneal injections of 50 μl of tamoxifen (Sigma) at 100 mg/kg in corn oil were given to induce expression of Ascl1 and GFP. Tamoxifen was administered once for P0 or P4 induction, for 2 consecutive days in mice aged P6-P9, or 4 consecutive days for older ages. Transgenic expression is detectable within 24 h.

### IHC and cell counts

For lineage tracing with *GFP* and *Ascl1* induction, animals were euthanized and the eyes removed for dissection and removal of the cornea and lens. Eyes were then fixed in 4% PFA in PBS for 1 h before being transferred to a 30% sucrose in PBS solution and kept overnight at 4 °C. Eyes were then frozen at − 80 °C in O.C.T. (Sakura Finetek) and sectioned to 14 μm by cryostat (Leica). Slides were incubated at room temperature in blocking solution (10% normal horse serum, 0.5% Triton X-100, in PBS) for 1 h. Slides were then incubated overnight at 4 °C in primary antibody diluted in blocking solution. Slides were then washed three times in PBS for 15 min each before a 1 h room temperature incubation in secondary antibodies (Life Technologies) in PBS. Slides were washed once before being incubated 5–10 min with 1:10,000 DAPI (Sigma) in the dark. At this point, slides were washed three times in PBS and coverslipped with Fluoromount-G (SouthernBiotech). Primary antibodies: goat anti-SOX2 (Santa Cruz, 1:500), mouse anti-HUC/D (Invitrogen, 1:100), chicken anti-GFP (Abcam, 1:500), goat anti-OTX2 (R&D Systems, 1:100), rabbit anti-RCVRN (Millipore, 1:1,000).

Section imaging was performed using an Olympus Fluoview confocal microscope, and random fields throughout the retina were sampled for cell counts. Cell types were identified and counted by localization within the retina, cell morphology, and marker co-staining for OTX2 and SOX9. Significance values between treatments were determined by one-way ANOVA with a post-hoc tukey test or by t-test.

### FACS^17^

Retinas were isolated via dissection away from surrounding tissues and then washed in PBS. Fluorescence was confirmed via live imaging under an inverted fluorescent microscope (Zeiss). Retinas were then incubated on a nutator in Papain and DNase I for 10 min at 37 °C. Retinas were then triturated to generate a single-cell suspension and transferred to a tube containing an equal volume of Ovomucoid. The suspension was then spun down at 300* g* for 10 min at 4 °C. Cells were resuspended in a solution of 100:1:1 Neurobasal:DNase:Ovomucoid, and passed through a 35 μm filter before sorting using a BD FACSAria III Cell Sorter (BD Biosciences). Gating was performed to isolate intact cells from debris and to isolate positive fluorescent glial or progenitor cells. Progenitor cells were isolated from SOX2 + Amacrine cells by removing the higher fluorescent neuronal population. Positive fractions containing fluorescently labelled MG were then spun down at 300* g* and resuspended for the appropriate assay.

### ATAC

Purified cells from live-cell FACS were input into a 15 μl transposase reaction with an input of 100 k cells in a protocol modified from the Greenleaf lab^41^. Transposition was carried out with reagents from the Nextera DNA Sample prep kit: 7.5 μl 2X TD Buffer, 0.75 μl Tn5 Transposases, and nuclease-free water to 15 μl. The reaction was mixed and incubated at 37 °C for one hour before being purified with the Qiagen Reaction Cleanup Minelute kit and eluted into 10 μl. Libraries were prepared through subsequent PCR using Illumina Nextera kit (Cat. No. FC-121–1,030) using a test qPCR output to estimate the number of cycles necessary to properly amplify the library. Amplified libraries were purified with the Qiagen PCR cleanup minelute kit and eluted into 20 μl. Library QC was performed using gel electrophoresis, and quantitated on a Qubit 3.0 Fluorometer with the dsDNA HS Assay kit and A260/280 and A260/230 checked by nanodrop before sending for Illumina NextGen sequencing on a Next Seq 500 in rapid mode employing a paired-end 50 base read length sequencing strategy (Seattle Genomics). Adapter and barcode sequence were removed from the reads and low-quality sequences (Phred score < 33) were removed using Trim Galore^42^. Remaining reads were mapped using Bowtie2^43^, marking duplicate reads with Picard (https://broadinstitute.github.io/picard/), and removing reads using Samtools^44^. Alignment data was normalized for coverage using a custom R script (https://rpubs.com/achitsaz/98857) and visualized using the Integrated Genomics Viewer^45^.

### Peak calling and comparison

Peaks were called using HOMER^46^ findPeaks dnase style with a minimum distance of 415 and size of 150. BEDOPS -e 1 and -n 1 functions were used to compare peak files for binary peak differences^14^. For differential accessibility comparisons, the R package EdgeR^47^ was used to compare all peak regions between two ATAC samples against the reads of each file as previously described. The RSubread^48^ function featureCounts was used to generate a matrix of counts per million across all peaks. The counts matrix was filtered against low-reads rows and in the case of any sample that is more deeply sequenced than another, the EdgeR^47^ function thincounts was used to thin one sample randomly to the level of the lower depth of sequencing. Dispersion, fitting and differential signal testing were performed using negative binomial generalized linear models as specified in the edgeR guide. Cumulative differences in accessibility at each gene were calculated as the sum of the fold differences of all peaks nearest to each gene. Peaks of interest were identified by selecting for those with a log2FC above 2, and the top 1 k peaks up and down were selected by log2CPM. The peakIDs for each of these regions were used to generate new peak files and perform further analysis.

### ASCL1 Chromatin Immunoprecipitation-Sequencing (ChIP-Seq)

P0 retinas or cultured, post-natal day 12, Müller glia (+ /- Ascl1 overexpression, rtTA germline:tetO-Ascl1-ires-GFP mice ± doxycycline) were digested with papain/DNase to single cells and fixed with 0.75% formaldehyde for 10 min at room temperature. Sonication was performed with a probe sonicator (Fisher Scientific): 12 pulses, 100 J/pulse, Amplitude: 45, 45 s cooling at 4 ºC between pulses. Immunoprecipitation performed with 40 mL anti-mouse IgG magnetic beads (Invitrogen Cat: 110.31) and 4 mg mouse anti-MASH1 antibody (BD Pharmingen Cat: 556,604) or 4 mg mouse IgG against chromatin from 5 million cells per condition according to Diagenode LowCell Number Kit using IP and Wash buffers as previously described^20^. Libraries were prepared with standard Illumina adaptors and sequenced to an approximate depth of 36 million reads each. Sequence reads (36 bp) were mapped to the mouse mm9 genome using bwa (v 0.7.12-r1039). Merging and sorting of sequencing reads from different lanes was performed with SAMtools (v1.2). The HOMER software suite was used to determine and score peak calls (‘findPeaks’ function, v4.7) as well as motif enrichment (‘findMotifs’ function, v4.7, using repeat mask). Reads were aligned to the mm9 genome using Bowtie2. The .sam files were converted to sorted .bam files using SAMtools. MACS2 was used to call peaks with default settings using the broad peaks annotation. Peak overlap analyses were performed using BEDOPS. The control ASCL1 ChIP-seq .bam file was downsampled by a factor of 0.69 to normalize the number of mapped reads over the common peaks found between treatment and control samples. This downsampled .bam file was used for all analyses. Differential accessibility analysis in ASCL1 ChIP-seq peaks was determined using edgeR as detailed in the edgeR user guide.

### Bulk RNA seq

For RNA-Seq, FAC-sorted cells were resuspended into Qiazol and RNA was extracted using the Qiagen RNeasy MinElute kit. Samples were tested for QC using nanodrop, and sent for sequencing. 500 ng per sample (50 ng μl^−1^) was sequenced on an Illumina HiSeq and reads that passed Illumina’s base call quality filter were mapped to mm10 using TopHat v2.0.12. To generate counts for each gene using htSeq-count v0.6.1p1, in “intersection-strict” overlap mode, genes with zero counts across all samples were removed, and data normalized using edgeR v3.12.0. Further analysis was done using Bioconductor and R (version 3.2.3).

EdgeR was used to compare reads across RNA-seq samples according to the published manual guide for exactTest^47^. Prior to testing, samples with higher reads were thinned to the level of other samples using thinCounts. Dispersion, fitting and differential signal testing were performed using negative binomial generalized linear models as specified in the edgeR guide. In our initial processing of RNA-seq data, all genes were used. For further analysis (4C and beyond), genes were sorted by logFC and the top 1,000 genes with a logFC > 2 for increasing expression were chosen, and vice versa for decreasing expression. This was done to select for only highly differentially expressed genes. The top genes were clustered using hclust to perform ward D2 agglomeration with Euclidian distances. Genes were filtered against annotated ATAC peaks by dplyr.

To compare bulk RNA-seq to previously published single cell RNA-seq in the retina, data from Clark et al. 2019 was subset to identify clusters of progenitors and MG^36^. Clusters were identified as MG or progenitors based on expression of markers *Slc1a3* and *Rlbp1*. Top gene expression in these clusters was identified using Seurat’s findmarkers function. Only genes with an adjusted p value of < 0.05 were considered for comparison.

### Cell culture and expression^27^

MG from postnatal day (P)12 mice were cultured [Neurobasal + N2, epidermal growth factor (EGF), 10% fetal bovine serum (FBS)] as previously described with 1 μM 5-ethynyl-2′-deoxyuridine (EdU). Lentiviral particles were added in Optimem (Gibco) or neural medium (Neurobasal + N2, B27, 1% tetracycline (tet)-free FBS), and 3–6 h later medium was replaced. hBDNF (R&D Systems, 10 ng/ml), bFGF (R&D Systems, 100 ng/ml) and rGDNF (R&D Systems, 10 ng/ml) were added for longer cultures. 4-Hydroxytamoxifen (4-OHT; Sigma) was included where indicated at 10 μM.

### Chi squared statistical testing

Accessibility genomic regions annotations were compared by chi-squared analysis with a posthoc test. Pearson’s Chi-Squared was performed with chisq.test() in R. Posthoc testing was performed using the chisq.posthoc.test package in R.

### Homer annotatePeaks.pl

For basic annotation of ATAC peaks, the Homer function annotatePeaks.pl was used^46^. While in most cases, peak annotation to genes was accomplished with the online tool GREAT^49^, annotating each region for closest gene, the basic usage was used:

annotatePeaks.pl [peak file] mm9 > output.txt.

For stats on genome location:

annotatePeaks.pl [peak file] mm9 -annStats > output_stats.txt.

For annotation of specific motifs, a position weight matrix was either taken from the homer database, from the findmotifs function, or was generated using seq2profile.pl in Homer, and was used in annotatePeaks as such:

annotatePeaks.pl [peak file] mm9 -m pwm.motif > output_motif.txt.

However, for the generation of lineplots, a table of 10 bp bins for motif enrichment were generated for graphing with ggplot2:

annotatePeaks.pl [peak file] mm9 -m pwm.motif -size 2000 -hist 10 > output_plot.txt.

For motif co-occurrence:

annotatepeaks.pl < peak file > mm9 -size 2000 -hist 20 -m < motifs of interest > -cpu 10 -matrix fileout > fileout.motif.freq.

### Homer motif discovery

To discover enrichment of predicted DNA binding motifs for further analysis, we employed the Homer function findMotifsGenome.pl using suggested basic usage settings. From there, we identified top motifs from the homerResults output.

### Gene ontology

For annotation of gene ontology (GO) categories, we used the Bioconductor package GOstats^50^. Gene lists from ATAC and RNAseq were input to a hyperGTest with a p value cutoff of 0.001 for Biological Process ontology categories. The top 20 GO terms were plotted in ggplot2. To acquire genes of interest from specific GO categories, we found annotations of the category ID on the Jax Mouse Genome Informatics database and subset our original genelist based on the genes in each GO category.

### Heat maps and plots (deeptools)

To generate heat maps and lineplots of ATAC and ChIP read enrichment, we used deeptools^51^ computeMatrix reference-point to calculate enrichment scores by region along a bed file: computeMatrix reference-point -S < bigwig files > -R < bed files > –referencePoint center -a 1,000 -b 1,000 –skipZeros – MAT_file.tab.gz.

This is then plotted with the command:

plotheatmap -m MAT_file.tab.gz -out HM_file.png –colorMap Blues –missingdatacolor 1.0.

Or, to plot the lineplots only:

plotProfile -m MAT_file.tab.gz -out Plot_file.png –yMax 3.0.

## Supplementary information

Supplementary Information

Supplementary Table S1

Supplementary Table S2

Supplementary Table S3

Supplementary Table S4

Supplementary Table S5

Supplementary Table S6

Supplementary Table S7

Supplementary Table S8

Supplementary Table S9

Supplementary Table S10

## Data Availability

All ATAC and RNA-seq datasets have been deposited in Gene Expression Omnibus with accession GSE137318.
