# Supplementary material for: Developmental changes in the accessible chromatin, transcriptome and Ascl1-binding correlate with the loss in Müller Glial regenerative potential
Source: Sci Rep. 2020 Aug 12;10:13615. doi: 10.1038/s41598-020-70334-1 (PMC7423883; doi:10.1038/s41598-020-70334-1)
Supplement: Supplementary file 1 — Supplementary Information [file 41598_2020_70334_MOESM1_ESM.pdf]

Supplementary Material for:

Developmental changes in the accessible chromatin, transcriptome and Ascl1-binding correlate with the loss in Müller Glial regenerative potential.

Leah S. VandenBosch[1,2], Stefanie G. Wohl[1,4], Matthew S. Wilken[1,2], Marcus Hooper[1], Connor Finkbeiner[1], Kristen Cox[1], Laura Chipman[1], Thomas A. Reh[1,3]*

1. Department of Biological Structure, University of Washington, Box 357420, Seattle, WA, 98195, USA.

2. Molecular and Cellular Biology Program, University of Washington, Seattle, WA, USA.

3. Institute for Stem Cells and Regenerative Medicine, University of Washington, Box 358056, Seattle, WA, 98109, USA.

4. Department of Biological and Vision Sciences, The State University of New York, College of Optometry, New York, NY, USA

*Corresponding Author tomreh@uw.edu

Supplementary Figures

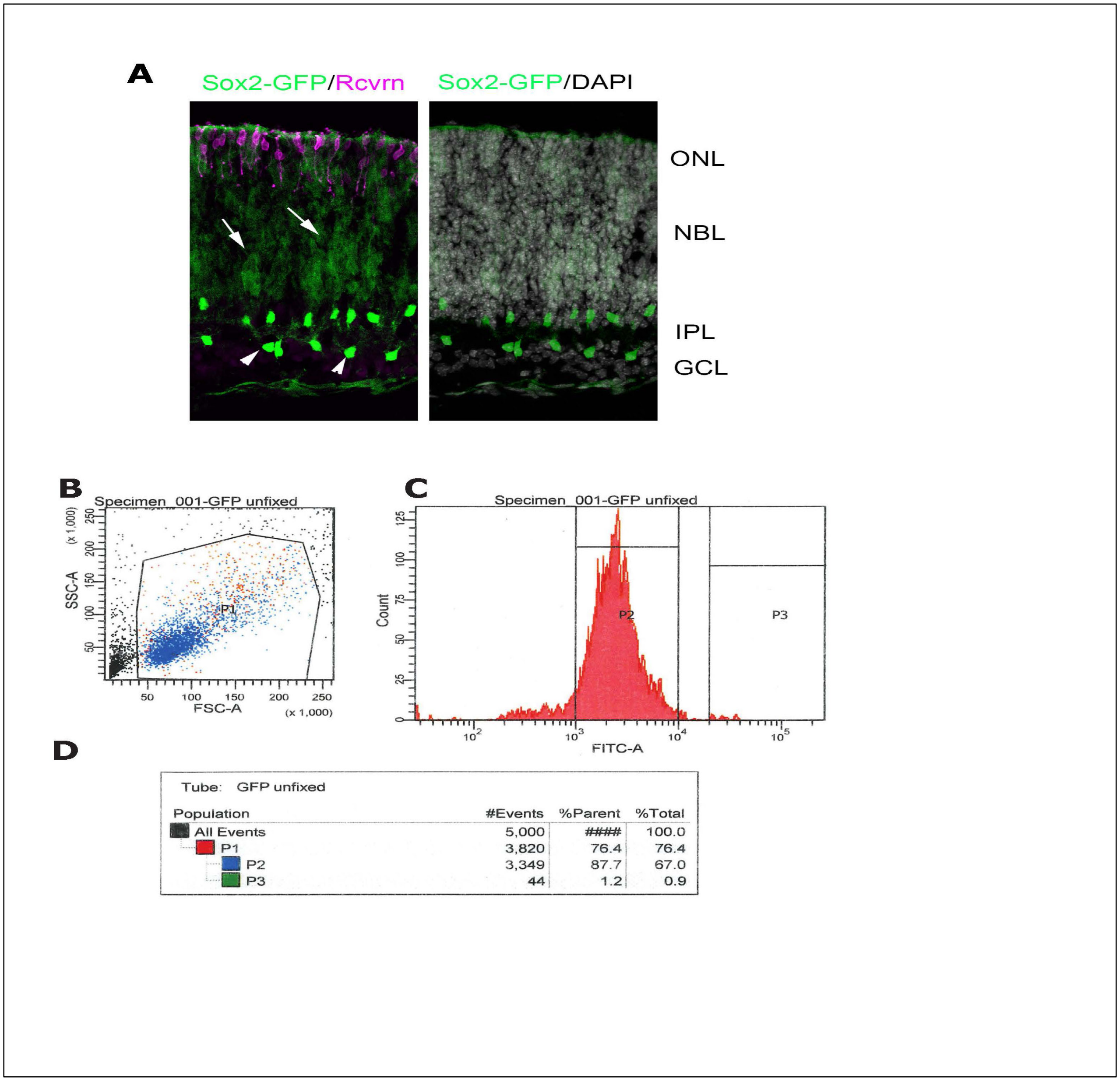

S1. Sox2 GFP allows purification of P2 progenitors from retina.

A. Imaging of Sox2GFP with Recoverin (left) and DAPI (right). Arrows identify low level GFP+ progenitor cells, arrowheads identify high level GFP+ starburst amacrine cells. B. FACS gating for P1 gate isolating whole cells. C. FACS gating for isolating individual cell populations. P2 gate selects mid-range GFP for progenitor cells, P3 gate selects high GFP amacrine cells. D. Breakdown of ratios of cell numbers for gating shown in B and C.

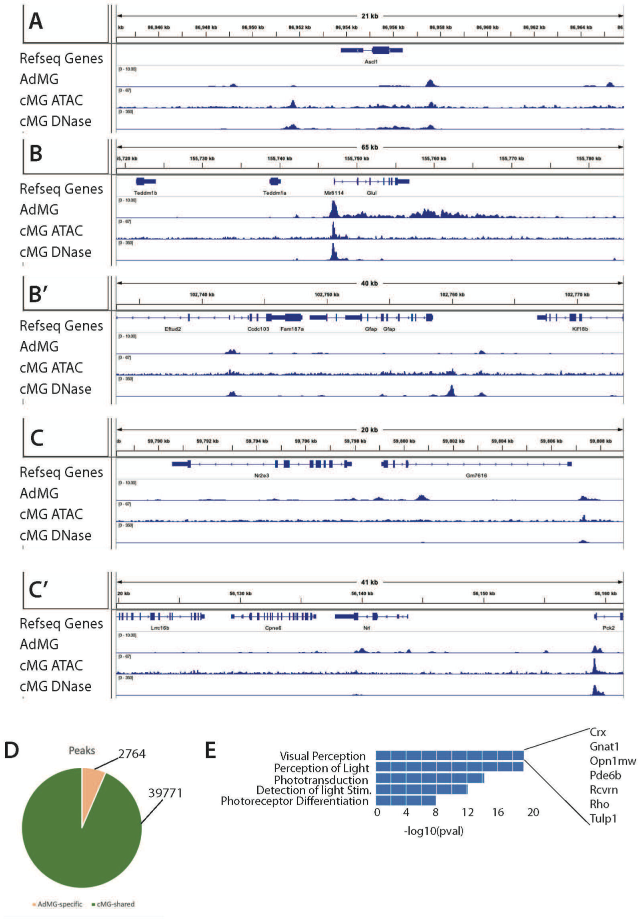

S2. AdMG compared with MG cells maintained in primary dissociated cell culture.

A-C’. Adult MG ATAC and cultured MG ATAC and DNase tracks for Ascl1 (A), glial genes Glul (B) and GFP (B’), and rod genes Nr2e3 (C) and Nrl (C’). D. Pie chart of overlaps of Adult MG ATAC Homer peaks with cultured MG DNAse Hotspots. E. Gene ontology by GProfiler of neighboring genes of peaks specific to Adult MG dataset and removed in overlap with cultured MG DNase data. Related genes in GO category of interest labeled to the right.

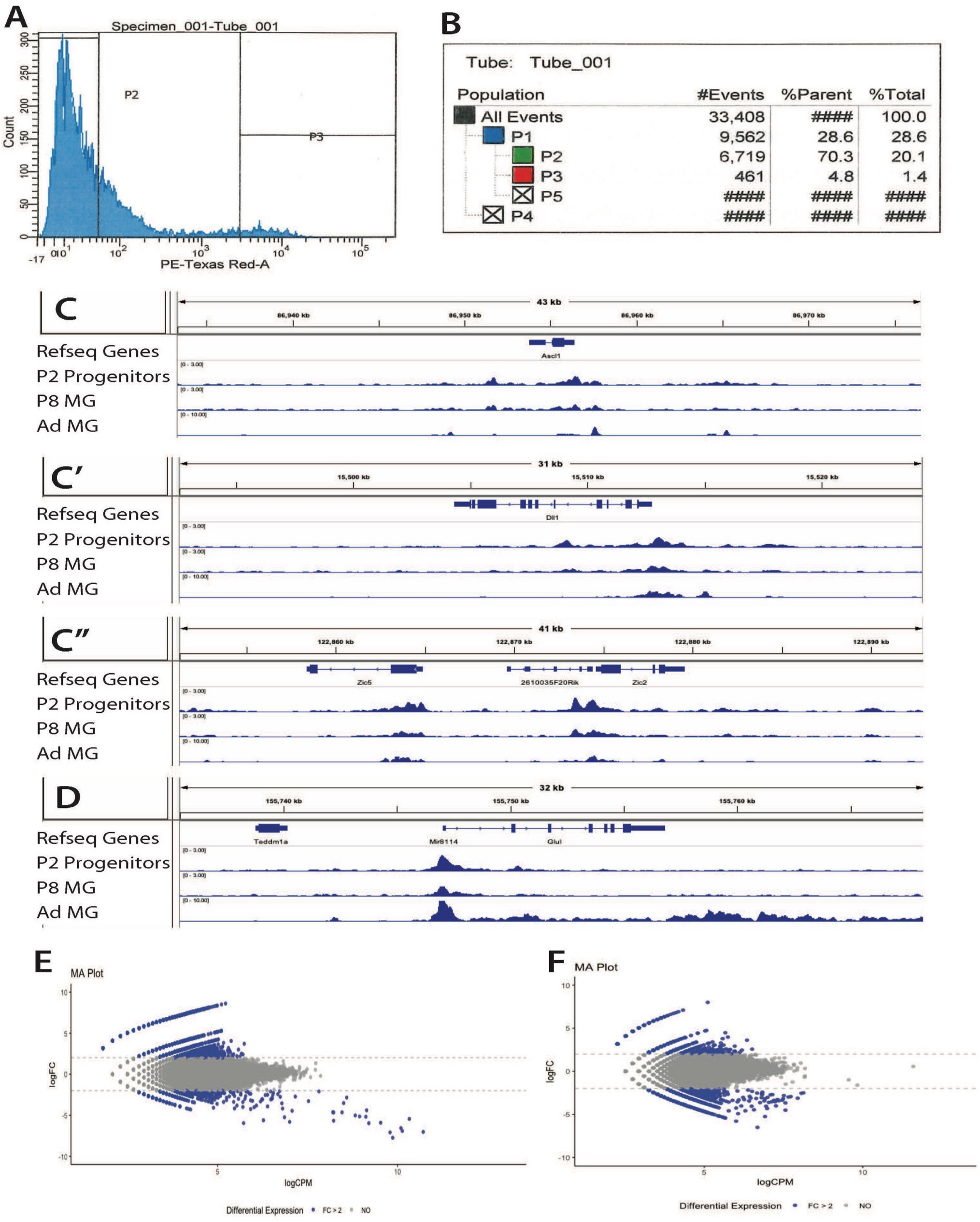

S3. P8 ATACseq results

A. FACS gating information for isolating separate populations. P2 gate on far right isolates TdTomato (-) cells, and P3 gate selects TdTomato (+) MG. B. Breakdown of ratios of cell numbers for gating shown in A. C-D. Tracks for P2 progenitor, P8 and Adult MG ATAC comparing reads for progenitor genes Ascl1 (C) Dll1 (C') and Zic2/5 (C'') and glial gene Glul (D). E-F. Differential Accessibility plots for P2 v. P8 (E) and P8 v. Adult MG (F).

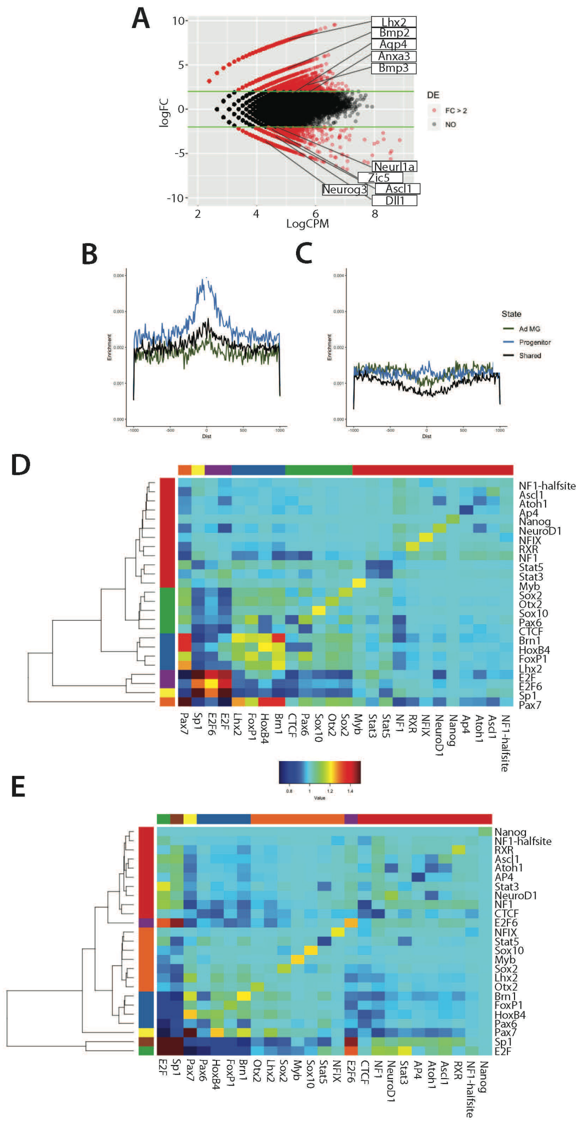

S4. Motif Enrichment examples.

A. Differential Accessibility plot of Progenitor ATAC-seq reads over adult MG ATAC-seq reads by EdgeR. Accessible domains in red differ between datasets by logFC>2 or <-2, where logFC>2 represents adult MG-enriched accessibility, and logFC < -2 represents Progenitor-enriched accessibility. Some peaks of interest labeled with neighboring genes annotated by GREAT. B-C. Lineplots of predicted central enrichment for (B)Ascl1- or (C) Neurog2-specific Ebox motifs in progenitor-specific, shared, or adult MG-specific accessible domains by Bedops D. Co-occurrence analysis by HOMER for top predicted motifs. Analysis shows likelihood of motifs occurring in the same accessible domain in progenitor-specific accessibility regions, hierarchical clustering of motifs by co-occurrence ratios. E. Co-enrichment matrix for adult MG-specific accessible domains.

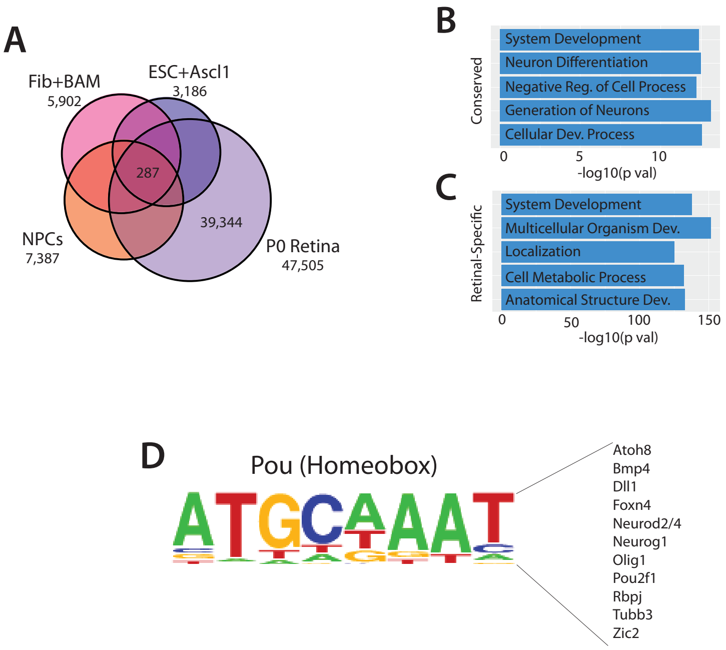

S5. Ascl1 ChIP in retinal progenitors compared with that from other cell types.

A. Venn diagram of overlaps between Ascl1 ChIP in whole P0 retina (RPCs) and published Ascl1 ChIP loci: Neural Progenitor cells (NPCs), Fibroblasts + BAM (Brn2, Ascl1, Myt1l) (Wapinski et al 2013), and Embryonic Stem cells (ESCs) + Ascl1 (Casey et al. 2018). Peak numbers indicated for each dataset, as well as intersection and retinal-specific peaks. B. Gene ontology by GProfiler for intersection of all datasets (conserved) and C. retinal-specific peaks. D. Venn diagram of overlaps by BEDOPS between P2 Progenitor ATAC and P0 Retina Ascl1 ChIP peaks. E. Pou motif found in common with overexpressed Ascl1 binding. Neighboring genes to Pou motifs listed to the right.

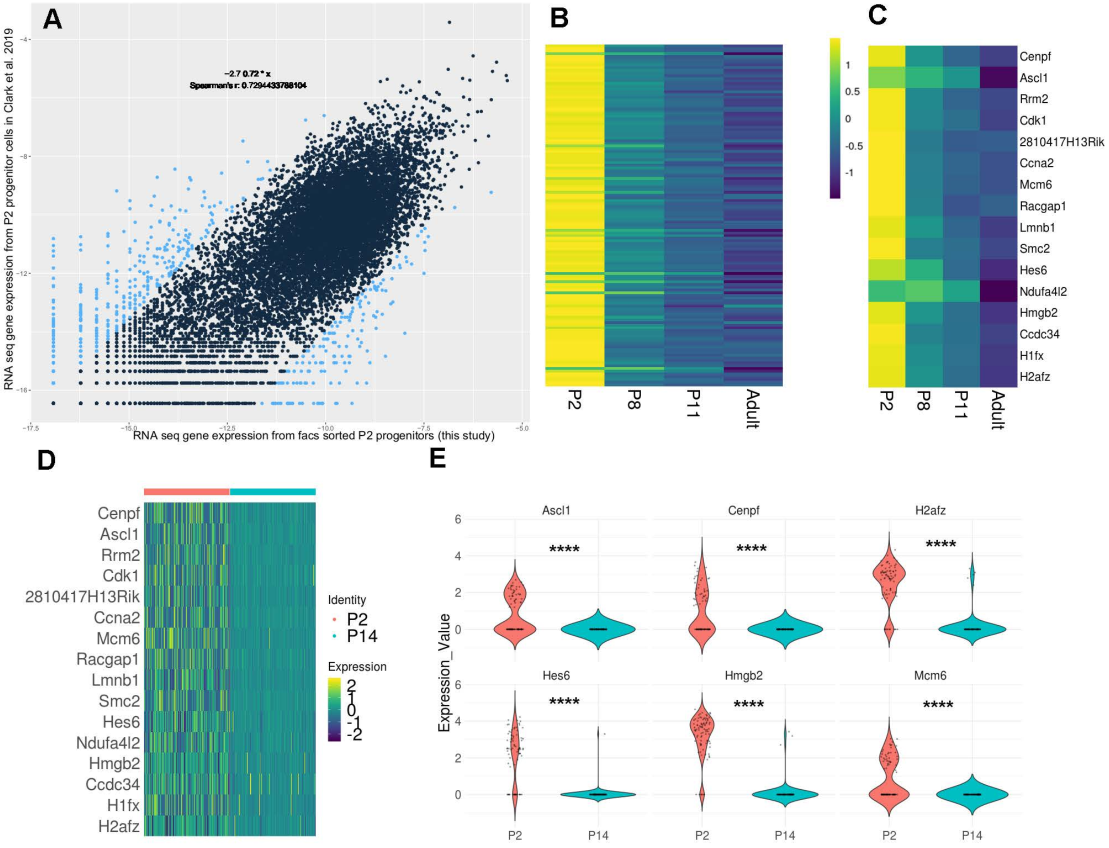

S6. RNA sequencing data from FACS purified progenitors is similar to single cell RNAseq data.

Data from Clark et. al 2019 were subset to include only P2 progenitors and P14 glia (defined by the subset of cells characterized by high expression of glial and progenitor markers Slc1a3 and Rlbp1). A: All markers of P2 sorted progenitors that were shared with single cell data from Clark et. al 2019. (B+C) Shown are a subset of markers that defined P2 progenitors in single cell RNA sequencing data from Clark et. al 2019. (B) data from this study (C) data from Clark et al. 2019. Many markers of P2 glia were cell cycle or cell differentiation genes. (D) Violin plots showing differential expression of marker genes between P2 progenitors and P14 glia. Statistics: Wilcoxon rank sum test comparing P2 vs P14, ****: $p<0.0001$.

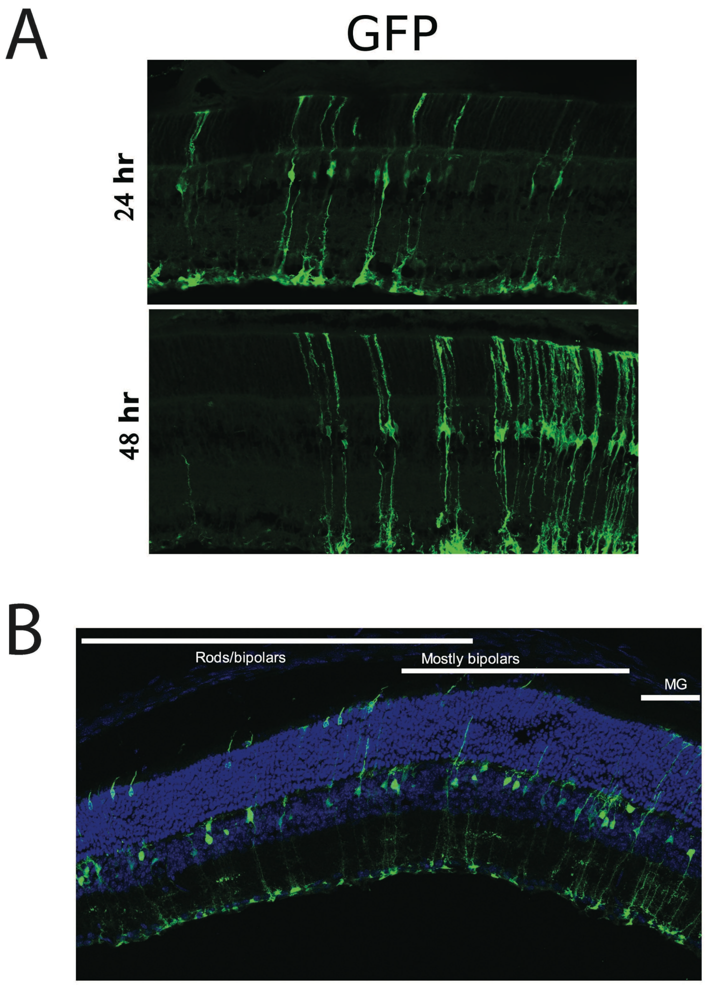

S7. Ascl1 overexpression in Immature Retinas

A. Transgenic GFP induction in Glast-CreER;LNL-tTA;Ascl1-ires-GFP mice 24 hours after Tamoxifen injection and 48 hours after induction. B. Fluorescent imaging of the peripheral retina with GFP cell tracing in Glast-CreER;flox-stop-ccGFP mouse line. Injections for cell tracing were done at P4, with retinas collected at P21 for histological analysis Cell identities labeled from far periphery left to central retina right. Scale bar 50um.

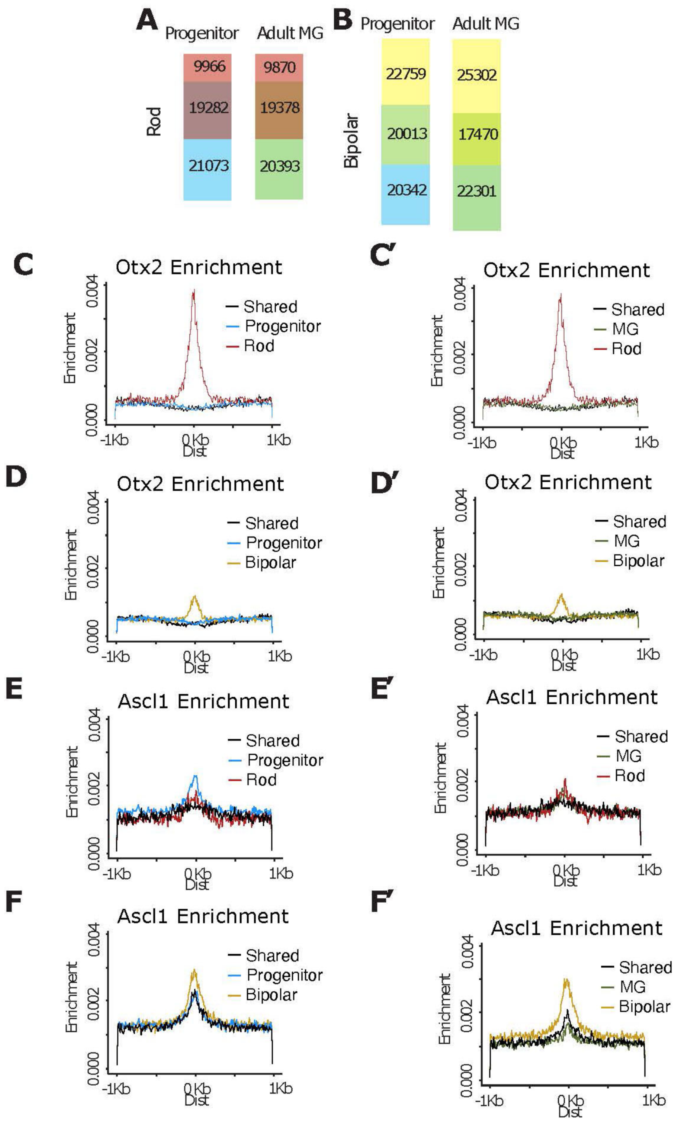

S8. Progenitor and MG Neuronal overlaps

A. Representation of peak number overlaps between Progenitors/Adult MG and Rod photoreceptors with progenitor peaks in blue, MG in green, and rod peaks in red. B. Representation of peak number overlaps between Progenitors/Adult MG and Bipolar cells with bipolar peaks in yellow, and progenitors/MG in same coloring as in A. C-F' Lineplots of central enrichment of Otx2 (C-D') and Ascl1 (E-F') in the overlaps represented in A and B. Progenitor overlaps are represented in C, D, E, and F, and MG overlaps are represented in C', D', E', and F'. Rod overlaps are represented in C-C' and E-E', and Bipolar overlaps are represented in D-D' and F-F'

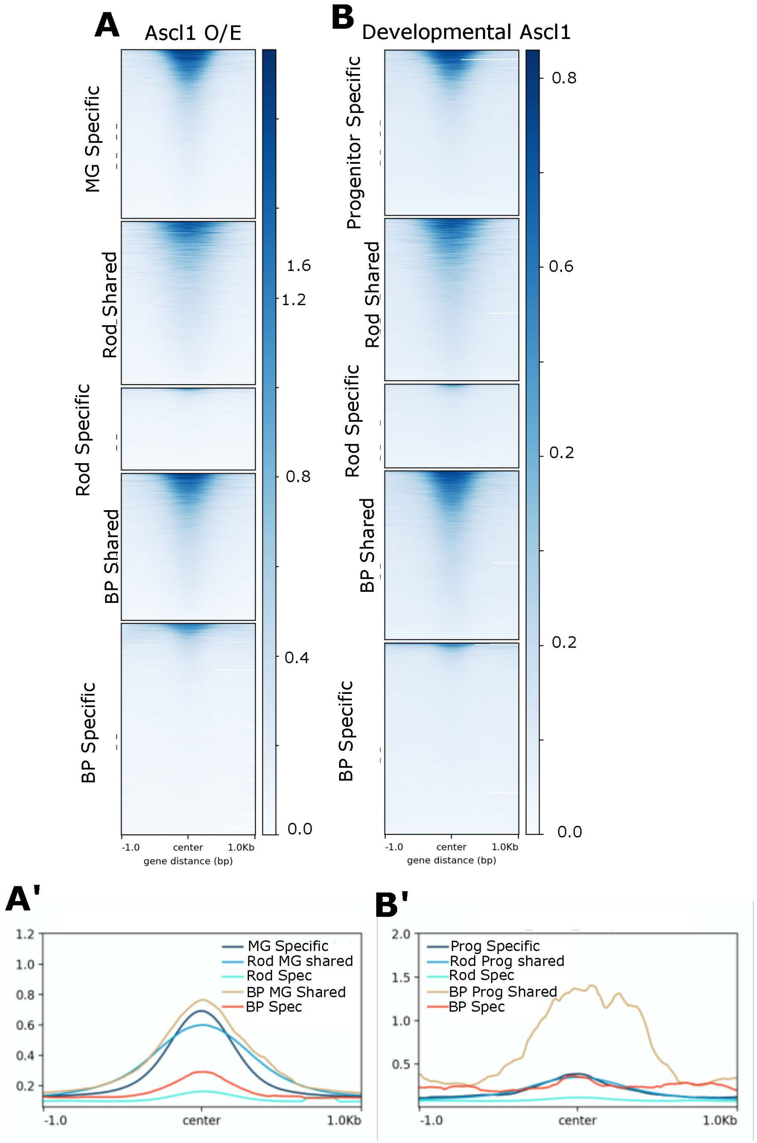

S9. Ascl1 binding in Accessible Neuronal Regions

A. Heat map and lineplot of Ascl1 binding in overexpression paradigm across regions of accessibility unique to MG, shared between MG and Rods or Bipolar cells, or unique to Rods or Bipolar cells. A'. Lineplot of Ascl1 binding across regions in A. B Heat map and lineplot of Ascl1 binding in P0 retinas across regions of accessibility unique to progenitors, shared between progenitors and Rods or Bipolar cells, or unique to Rods or Bipolar cells. B'. Lineplot of Ascl1 binding across regions in B.

Supplementary Tables

Table S1 ATAC Gene Ontology

Table S2 Motifs Table

Table S3 Neurogenic related genes

Table S4 RNA-seq genelists by cluster

Table S5 RNA-seq GO terms by cluster

Table S6 RNA-seq and ATAC-seq overlaps genes

Table S7 RNA- and ATAC-seq overlaps Gene Ontology

Table S8 Neuron Overlaps GO tables
